# The production of human glucocerebrosidase in glyco‐engineered *Nicotiana benthamiana* plants

**DOI:** 10.1111/pbi.12529

**Published:** 2016-02-12

**Authors:** Juthamard Limkul, Sayoko Iizuka, Yohei Sato, Ryo Misaki, Takao Ohashi, Toya Ohashi, Kazuhito Fujiyama

**Affiliations:** ^1^ International Center for BiotechnologyOsaka UniversitySuita‐shiOsakaJapan; ^2^ Division of Gene TherapyResearch Center for Medical SciencesThe Jikei University School of MedicineMinato‐kuTokyoJapan

**Keywords:** human glucocerebrosidase, *N*‐acetylglucosaminyltransferase I, glyco‐engineered plant, *Nicotiana benthamiana*, plant‐made pharmaceuticals

## Abstract

For the production of therapeutic proteins in plants, the presence of β1,2‐xylose and core α1,3‐fucose on plants’ *N*‐glycan structures has been debated for their antigenic activity. In this study, RNA interference (RNAi) technology was used to down‐regulate the endogenous *N*‐acetylglucosaminyltransferase I (GNTI) expression in *Nicotiana benthamiana*. One glyco‐engineered line (*Nb* GNTI‐RNAi) showed a strong reduction of plant‐specific *N*‐glycans, with the result that as much as 90.9% of the total *N*‐glycans were of high‐mannose type. Therefore, this *Nb* GNTI‐RNAi would be a promising system for the production of therapeutic glycoproteins in plants. The *Nb* GNTI‐RNAi plant was cross‐pollinated with transgenic *N. benthamiana* expressing human glucocerebrosidase (GC). The recombinant GC, which has been used for enzyme replacement therapy in patients with Gaucher's disease, requires terminal mannose for its therapeutic efficacy. The *N*‐glycan structures that were presented on all of the four occupied *N*‐glycosylation sites of recombinant GC in *Nb* GNTI‐RNAi plants (GC ^*gnt1*^) showed that the majority (ranging from 73.3% up to 85.5%) of the *N*‐glycans had mannose‐type structures lacking potential immunogenic β1,2‐xylose and α1,3‐fucose epitopes. Moreover, GC ^*gnt1*^ could be taken up into the macrophage cells via mannose receptors, and distributed and taken up into the liver and spleen, the target organs in the treatment of Gaucher's disease. Notably, the *Nb* GNTI‐RNAi line, producing GC, was stable and the *Nb* GNTI‐RNAi plants were viable and did not show any obvious phenotype. Therefore, it would provide a robust tool for the production of GC with customized *N*‐glycan structures.

## Introduction

Plant‐made pharmaceuticals (PMPs) are of interest due to their significantly lower cost of production compared to the more widely used animal cell‐cultured systems. In addition, plants are free from animal‐specific viral pathogen contaminations, which are a serious concern for biopharmaceutical productions. However, the presence of core β1,2‐xylose and α1,3‐fucose in the *N*‐glycan structures produced in plants (Fitchette‐Laine *et al*., 1994; Strasser, 2014), which differ from the *N*‐glycan structures produced in humans, is one of the major obstacles to the practical use of plants for pharmaceutical glycoprotein production. Over the past two decades, many studies have attempted to modify plant *N*‐glycosylation in order to overcome the limitations to safety, quality and efficacy of the PMP products resulting from the plant *N*‐glycan structures (Yoo *et al*., 2014).

*N*‐glycosylation processes in the endoplasmic reticulum (ER) of plants are similar to those in humans, but the modification processes in the Golgi complex differ between plants and humans, with the former leading to the formation of plant‐specific sugars that may induce immune responses in humans (Altmann, 2007; Bosch *et al*., 2013; van Ree *et al*., 2000). The *N*‐acetylglucosaminyltransferase I (GNTI) localized in the *cis*‐Golgi is the first enzyme in the pathway for biosynthesis of hybrid and complex *N*‐glycans, and catalyses the addition of *N*‐acetylglucosamine to the Man5GlcNAc2 structure (Dohi *et al*., 2010). The deficiency in GNTI activity of *Arabidopsis thaliana complex‐glycan‐deficient* (*cgl*) mutants results in the absence of complex *N*‐glycans on endogenous glycoproteins and a drastic increase in high‐mannose forms, predominantly the Man5GlcNAc2 structure (von Schaewen *et al*., 1993; Strasser *et al*., 2005). Moreover, stable antisense suppression of *GNTI* in potato (*Solanum tuberosum* L.) and tobacco (*Nicotiana tabacum* L.) was also shown to result in a substantial reduction of complex glycan patterns (Wenderoth and von Schaewen, 2000). However, the down‐regulation of GNTI in a tobacco‐related species, *Nicotiana benthamiana*, showed a reduction of more than 85% in *in vitro* enzyme assays but no significant changes in the total *N*‐glycan profiling versus the wild‐type plants, as reported by Strasser *et al*. (2004).

Gaucher's disease is a lysosomal storage disorder caused by mutations of the glucocerebrosidase (GC) gene located on chromosome 1 (Ginns *et al*., 1985) which result in the progressive accumulation of glucocerebroside in the lysosomes of macrophages in several visceral organs. Type I Gaucher's disease is currently treated by enzyme replacement therapy (ERT). The commercial recombinant GC for ERT has been produced in various cells including Chinese hamster ovary (CHO) cells, with the resulting enzyme marketed as imiglucerase (Cerezyme^®^; Genzyme Corp., Cambridge, MA); cultured human cells, with the product marketed as velaglucerase alfa (VPRIV^®^; Shire Plc, St Helier, Jersey); and cultured carrot cells, with the product marketed as taliglucerase alfa (Elelyso^®^; Protalix BioTherapeutics, Inc.**,** Carmiel, Israel and Pfizer Inc., New York City, NY), which is the first plant cell‐based ERT approved by the US Food and Drug Administration (FDA). GC is a glycoprotein and its terminal mannose residues on the *N*‐glycans play an important role in its targeting to macrophages, where glucocerebroside accumulates, and in the internalization via mannose receptor. Recently, the recombinant plant‐derived GC (taliglucerase alfa) expressed in carrot cells targeted to the storage vacuoles was shown to naturally display terminal mannose residues on its glycans (Shaaltiel *et al*., 2007). Unlike the GC produced in CHO cells, the recombinant plant‐derived GC does not require *in vitro* enzymatic modification to expose its mannose residues. However, it still contains plant‐specific *N*‐glycans, core β‐1,2‐xylose and α‐1,3‐fucose residues. He *et al*. (2012) reported an active GC produced in the seeds of Arabidopsis *cgl* mutants mainly contained mannose‐type *N*‐glycans; however, the low biomass of Arabidopsis seeds might be a drawback in terms of large scale production. The *N. benthamiana* plant could be an economical alternative production system because it has already been used successfully to express various pharmaceutical proteins (Klimyuk *et al*., 2014; O'Keefe *et al*., 2009; Ramírez *et al*., 2002; Strasser *et al*., 2008). We recently reported that the combination of the 5′ untranslated region of the Arabidopsis alcohol dehydrogenase gene with the Arabidopsis heat shock protein terminator provided a high yield of human GC in *N. benthamiana* plants, that is 68 μg protein/g fresh weight of leaves, which was estimated to be 1.45% total soluble protein (Limkul *et al*., 2015).

In this study, we modulated the *N*‐glycan composition in *N. benthamiana* using an RNA interference (RNAi) strategy to obtain a targeted down‐regulation of endogenous GNTI expression. This glycosylation mutant was cross‐pollinated with an *N. benthamiana‐*expressed GC mutant to produce GC that mainly carried the Man5GlcNAc2 structure, which is beneficial not only for macrophage targeting but also for safety from plant‐specific epitopes. An *in vitro* study using macrophage cells confirmed that GC^*gnt1*^ could be taken up via mannose receptors and an *in vivo* study demonstrated that it could be taken up into the target organs.

## Materials and methods

### Plasmid construction

A *GNTI*‐coding sequence was amplified from cDNA of *Nicotiana tabaccum* as described in Dohi *et al*. (2010). DNA fragments containing sense (nt. 460–1341) and antisense (nt. 834–1341) sequences were amplified by PCR from the NtGNTI cDNA using the forward primer *Bam*HI‐GNTIsense_F (5′‐GGGGATCCGCTTTGAGCTATGATCAGCT‐3′), reverse primer *Sac*I‐GNTIsense_R (5′‐TAGAGCTCTTAAGTATCTTCATTTCCGA‐3′), *Bam*HI‐GNTIantisense_F (5′‐ACGGATCCCAAAGTGGCCAAAGGCTTAC‐3′) and *Xba*I‐GNTIantisense_R (5′‐TATCTAGATTAAGTATCTTCATTTCCGA‐3′). The antisense and sense fragments were fused by ligation (*Bam*HI site) and were assembled onto a complementary sticky end of the pGPTV‐HPT backbone (*Xba*I and *Sac*I sites), which had been modified with a 35S terminator derived from the Cauliflower mosaic virus (CaMV). The GNTI‐RNAi fragment was driven under control of the CaMV 35S promoter. The plasmid construct was verified by sequencing.

### Stable transformation of *Nicotiana benthamiana* plants

The *N. benthamiana* transformation protocol was adapted from Liu *et al*. (1994). Briefly, leaf discs of wild‐type *N. benthamiana* plants were co‐cultivated with *Agrobacterium* carrying pGNTI‐RNAi for 3 days and then transferred onto Murashige and Skoog (MS) medium supplemented with 1 mg/L benzyl aminopurine (BA), 0.1 mg/L 1‐naphthaleneacetic acid, 250 mg/L carbenicillin and 30 mg/L hygromycin for selection. The leaf discs with developed shoots were transferred onto MS medium supplemented with 0.1 mg/L BA for bud elongation. Then, an individual shoot was separated and transferred onto hormone free medium with a selective antibiotic for root generation. The regenerated plantlets (designated the T_0_ generation) were transferred to soil and maintained under greenhouse conditions. The seeds of individual T_0_ plants were harvested and used to germinate next‐generation plantlets by selection on MS medium containing hygromycin. The transgenic plants were repeatedly allowed to self‐fertilize, grown to seeds and then plated on a selective medium to generate T_2_, T_3_, T_4_ and T_5_.

### Generation of a GNTI‐knockdown *Nicotiana benthamiana*‐derived glucocerebrosidase

The stable GC‐expressing *N. benthamiana* plant (At‐GC‐HSP19) was generated as we described previously in Limkul *et al*. (2015). *Nb*GNTI‐RNAi7 (T_5_ generation) was cross*‐*pollinated with pollen from At‐GC‐HSP19 (T_5_ generation). The seeds produced from cross‐pollination were sown on selective medium containing 30 mg/L hygromycin (for selection of GNTI‐RNAi) and 10 mg/L bialaphos (for selection of GC). The germinated T_1_ generation plantlets were transferred to a greenhouse, and protein was extracted from the individual line for immunoblotting analysis and activity assay. The seeds from the selected line (*Nb*GC^*gnt1*^16) were germinated on selective medium to produce the T_2_ generation used for protein purification and glycan analysis.

### Glucocerebrosidase enzymatic activity assay

Measurement of the GC activities was carried out as described previously (He *et al*., 2012). The mixture of protein extract, activity assay buffer (1.3 mm EDTA, 0.125% sodium taurocholate, 4 mm β‐mercaptoethanol, 0.15% Triton X‐100, 60 mm phosphate‐citrate buffer, pH 6.0) and 4‐methylumbelliferyl β‐d‐glucopyranoside (4‐MUGP) was incubated at 37 °C for 1 h. After incubation, the reaction was terminated by glycine buffer (0.2 m glycine, 0.125 m sodium carbonate, pH 10.7). Fluorescence of the reaction product, 4‐methylumbellriferone (4‐MU), was monitored using an F‐2500 fluorescence spectrophotometer (Hitachi, Tokyo, Japan) (λ_excitation_ = 365 nm, λ_emission_ = 460 nm). One unit (U) of activity was defined as the amount of enzyme required to release 1 nmole 4‐MU/min. The specific activity is the units per mg of total soluble protein.

### SDS‐PAGE and immunoblotting analysis

Proteins extracted from the leaves of the 2‐month‐old transgenic plants were loaded onto 7.5%, 10% or 5%–20% sodium dodecyl sulphate‐polyacrylamide gel electrophoresis (SDS‐PAGE). After electrophoresis, the proteins were transferred onto a polyvinylidene difluoride (PVDF) membrane (Millipore*,* Billerica*,* MA). The membrane was blocked with 5% skim milk in PBS/T (1.47 mm KH_2_PO_4_, 10 mm Na_2_HPO_4_, 2.7 mm KCl, 137 mm NaCl, 0.05% Tween‐20, pH 7.4) for 30 min. The polyclonal antiglucocerebrosidase (GC) antibody (from rabbit; Sigma‐Aldrich, St. Louis, MO) diluted at 1 : 10 000 in PBS/T or polyclonal antihorseradish peroxidase (HRP) antibody (from rabbit; Sigma‐Aldrich) diluted at 1 : 10 000 in PBS/T was incubated with the membrane for 1 h. The anti‐rabbit IgG, HRP‐linked whole antibody (from donkey; GE Healthcare, Tokyo, Japan) diluted at 1 : 20 000 in PBS/T was allowed to bind the primary antibody for 1 h. For detection, the membrane was incubated with Luminata™ Forte Western HRP substrate (Millipore) for 5 min at room temperature and was detected by exposing the membrane to X‐ray film (Fujifilm Corporation, Tokyo, Japan). For Figure 3, the membrane was first detected with anti‐HRP antibody, and then, it was stripped using WB Stripping Solution (Nacalai Tesque, Kyoto, Japan) for 1 h at room temperature. After washing with PBS/T, the membrane was reblotted with anti‐GC antibody and was detected as described earlier. The proteins in polyacrylamide gels were stained following the manufacturer's instructions using a Silver stain II kit (Wako Pure Chemicals, Osaka, Japan) or Coomassie Brilliant Blue (CBB) Stain One (Ready To Use; Nacalai Tesque).

### *N*‐Glycans preparation and structural analysis using RP‐HPLC and LC–MS/MS

*N*‐glycans were released from glycoproteins extracted from the leaves of the 2‐month‐old wild‐type and *Nb*GNTI‐RNAi7 plants by hydrazinolysis at 100 °C for 10 h and then labelled with 2‐aminopyridine (PA) as described previously (Misaki *et al*., 2001). The PA‐labelled glycans were purified using cellulose‐column chromatography (Shimizu *et al*., 2001), and the excess PA reagents were removed using a MonoSpin NH2 desalting column (GL Sciences, Tokyo, Japan). The purified PA‐labelled glycans were injected into a reverse phase (RP)‐HPLC system (Elite LaChrom HPLC System; Hitachi) using a Cosmosil 5C18‐AR‐II column (6.0 × 250 mm; Nacalai Tesque). The mobile phase for RP‐HPLC was composed of solvent A (0.02% trifluoroacetic acid—TFA) and solvent B (20% acetonitrile/0.02% TFA). RP‐HPLC was performed at a flow rate of 1.2 mL/min and increased the percentage of solvent B linearly from 0% to 20% over 35 min. The eluted fractions were monitored by fluorescence intensity at an excitation wavelength of 310 nm and emission wavelength of 380 nm. The eluted fractions collected from RP‐HPLC were further analysed using an LC–MS/MS system (Agilent Technologies, Santa Clara, CA) equipped with HCT plus software (Bruker Daltonics, Billerica, MA). The mobile phase for LC was composed of solvent C (2% acetic acid in acetonitrile) and solvent D (3% triethylamine/5% acetic acid in water). The LC was performed using a Shodex Asahipak NH2P‐50 2D column (2.0 × 150 mm; Showa Denko, Tokyo, Japan) at a flow rate of 0.2 mL/min, and the percentage of solvent D was increased linearly from 20% to 55% over 35 min. The operating parameters for MS/MS were set as follows: positive‐ion mode, mass range 350–2750 m/z, nebulizer flow 5.0 psi, dry gas flow rate 3.0 L/min, dry temperature 300 °C, target count 200 000 and MS/MS Frag. Ampl. 1.0 V. The amount of *N*‐glycans was quantified by the peak area in LC.

### Glucocerebrosidase purification

The purification protocol was developed based on He *et al*. (2012) and Shaaltiel *et al*. (2007). Leaves of the 2‐month‐old At‐GC‐HSP19 or *Nb*GC^*gnt1*^16 transgenic plants were ground in liquid N_2_ and proteins were extracted with the extraction buffer containing 20 mm Tris, pH 7.0, 150 mm NaCl, 0.5% taurocholic acid and 1 mm PMSF. Following incubation in 4 °C for 20 min and centrifugation at 10 000 ***g*** for 20 min, the supernatant was applied to a Con A‐Agarose column (J‐oil mills, Tokyo, Japan) using a peristaltic pump (ATTO, Osaka, Japan) with recycling for 24 h at 4 °C. After washing the column, the glycoproteins were eluted with elution buffer (300 mm methyl‐α‐mannoside in the GC activity assay buffer) by recycling the buffer for 30 min, then collected and added to fresh elution buffer. The elution step was repeated 6–8 times. The eluant was concentrated using a Vivaspin column with a 50 kDa cut‐off membrane (Sartorius, Gottingen, Germany) and the buffer was exchanged with 2 m NaCl in 50 mm phosphate buffer, pH 7.0 (buffer A). The concentrated eluant was diluted in buffer A (1 : 5) and subjected to a hydrophobic interaction resin (Toyopearl Phenyl‐650C; Tosoh Corp., Tokyo, Japan) that had been pre‐equilibrated with buffer A. The elution was performed by decreasing the concentration of NaCl and increasing the concentration of ethylene glycol. The eluted fractions were assayed for GC activity, and the active fractions were combined and concentrated using a Vivaspin column with a 50 kDa cut‐off membrane. The purified GC was stored in 0.1 m acetate buffer, pH 6.0 containing 25% glycerol at −80 °C. The protein concentration was determined using the linearization of the Bradford protein assay (Ernst and Zor, 2010; Zor and Selinger, 1996).

### Deglycosylation with EndoH_f_ or PNGaseF

For endoglycosidase H (EndoH_f_) treatment, 100 ng of purified GC^WT^ and GC^*gnt1*^ were denatured in glycoprotein denaturing buffer by heating at 100 °C for 10 min. The denatured mixture was then treated with GlycoBuffer, water and EndoH_f_ (New England Biolabs, Ipswich, MA) and incubated at 37 °C for 1 h. For peptide‐*N*‐glycosidase F (PNGaseF) treatment, the samples were denatured in denaturing buffer with 0.2 m β‐mercaptoethanol by heating at 100 °C for 10 min, followed by the addition of stabilizer solution, water and PNGaseF (Takara Bio Inc., Kyoto, Japan) and incubation at 37 °C over‐night. At the end of the reaction, the samples treated with EndoH_f_ and PNGaseF and the nontreated samples were separated by SDS‐PAGE on 7.5% acrylamide gel (Wako Pure Chemicals) and subjected to immunoblotting analysis using anti‐GC antibody.

### Analysis of *N*‐glycans attached to glucocerebrosidase expressed in wild‐type and down‐regulated GNTI *N. benthamiana* plants

The purified GC^WT^ and GC^*gnt1*^ (2 μg) were separated on SDS‐PAGE and stained with CBB. The GC bands were excised from the gel following reduction and alkylation as described previously (Morelle and Michalski, 2007). The proteins were digested in the gel using trypsin Gold (Promega, Madison, WI) in ProteaseMAX™ surfactant (Promega) at 50 °C for 1 h. The peptides were extracted from the gel slices with 1% TFA in 60% acetonitrile and dried. The samples were dissolved in 0.1% formic acid. LC‐MS/MS analyses were performed on an ESI‐Qq‐TOF mass spectrometer (micrOTOF‐Q II; Bruker Daltonics) using a nano LC system (1200 series; Agilent Technologies) incorporating a trap column (5 μm, 0.3 × 5 mm) and analytical column (3.5 μm, 0.075 × 150 mm), both packed with Zorbax 300SB C‐18. For the nano LC system, the mobile phase consisted of 0.1% formic acid in water (solvent E) and 0.1% formic acid in acetonitrile (solvent F). The tryptic peptides were trapped in the column at a flow rate of 10 μL/min for 5 min. Elution was performed at a flow rate of 0.6 μL/min using a 2% to 8% gradient of solvent F over 5 min followed by a linear increase of solvent F to 50% for 40 min at 35 °C. After elution, the column was washed with 95% solvent F for 5 min before returning to the initial conditions. For MS and MS/MS analyses, the system was operated with automatic switching between MS and MS/MS modes. The operating parameters were set as follows: positive‐ion mode, mass range 50–4500 m/z, nebulizer flow 1.0 psi, dry gas flow rate 5.0 L/min, dry temperature 180 °C, and ISCID energy 5.0 eV. The three most abundant signals (absolute threshold >20 counts/s) were selected on each MS spectrum for further isolation and fragmentation. The complete system was fully controlled by micrOTOF control software (Bruker Daltonics). Bruker Compass DataAnalysis (version 4.0) was used for glycan analysis and Biotools (version 3.2) was used for *de novo* sequencing.

### GC^WT^ or GC^*gnt1*^ uptake by macrophages

Macrophage cells were obtained from mice as described in Gregory (1988). Briefly, bone marrow cells were collected from the femora of C57BL/6J, then washed and resuspended in 50% Dulbecco's modified Eagle's medium (DMEM) containing 10% heat‐inactivated foetal bovine serum (FBS), 20% heat‐inactivated horse serum (HS), 20% L‐929 cell‐conditioned medium (CM), 100 U/mL penicillin and 200 μg/mL glutamine. The macrophage cells were cultured at 37 °C in a humidified atmosphere composed of 5% CO_2_ in air. To confirm the purity of macrophages, cells were incubated first with mouse BD Fc‐Block (BD Pharmingen™; BD Biosciences, Franklin Lakes, NJ) and stained with the anti‐mouse CD11b PE and analysed on a flowcytometer (MACSQuant^®^; Miltenyi Biotec, Bergisch Gladbach, Germany). The cells were plated at 2 × 10^5^ cells/well in 24‐well plates, and incubated at 37 °C for 3 h in culture medium exhibiting equal levels of activity of GC^WT^ or GC^*gnt1*^ (90 units) with or without mannan (Sigma‐Aldrich). The mock was treated with phosphate‐buffered saline (PBS) used as a negative control. The medium was subsequently removed, and the cells were washed three times with PBS. Adherent cells were collected, and sonicated in lysis buffer containing 50 mm phosphate buffer, pH 6.5 and 0.25% Triton X‐100. The amounts of GC^WT^ or GC^*gnt1*^ taken up by the cells were determined by enzymatic activity assay. The equal levels of 45 units activity with or without mannan were used for the comparison with commercial GC (Cerezyme^®^).

### Biodistribution of GC^WT^ and GC^*gnt1*^ into organs

The wild‐type C57BL/6J mice used in this study were purchased from Sankyo Labo Service (Tokyo, Japan). All animal experiments were reviewed and approved by the Animal Care Committee of The Jikei University School of Medicine. A single infusion of either recombinant GC was performed according to the method described by Tekoah *et al*. (2013). GC^WT^ and GC^*gnt1*^ were administered into one of the tail veins of the C57BL/6J mice by single bolus injections at 90 units/g. The mock was injected with PBS used as a negative control. Groups of three rodents per enzyme were sacrificed at 60 min after injection. The spleen, liver, lung and kidney were collected and frozen at −80 °C until use. The organs were lysed with extraction buffer containing 20 mm phosphate buffer, pH 7.2, 20 mm EDTA, 20 mm l‐ascorbic acid and 1% Triton X‐100, and then assessed for enzymatic activity.

## Results

### RNAi‐mediated silencing of β1,2‐*N*‐acetylglucosaminyltransferase I (GNTI) is generated in *Nicotiana benthamiana*

**Figure 1:**
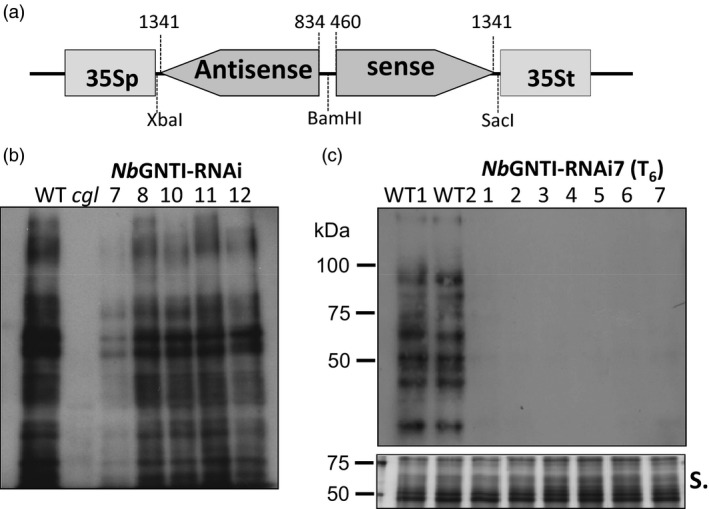
Generation of GNTI suppression in *Nicotiana benthamiana* plants. (a) Schematic representation of a construct used to generate GNTI suppression in *N. benthamiana* plants. 35Sp and 35St represent sequences of the cauliflower mosaic virus 35S promoter and terminator sequences, respectively. Antisense and sense sequences were derived from a coding mRNA sequence of *Nicotiana tabacum* β‐1,2‐*N*‐acetylglucosaminyltransferase. (b–c) Protein extracts were separated by 10% SDS‐PAGE and detected by immunoblotting using an antihorseradish peroxidase (anti‐HRP) antibody specific for plant complex‐type *N*‐glycans. (b) The *Nb* GNTI‐RNAi transgenic lines 7, 8, 10, 11 and 12 referred to an independent GNTI suppression transgenic line of T_2_ generation *N. benthamiana* plants. WT,*N. benthamiana* wild‐type plant; *cgl*,*Arabidopsis thaliana complex‐glycan‐deficient* plant. (c) The seven independent transgenic plants of *Nb* GNTI‐RNAi7 (T_6_ generation) and two independent WT plants (WT1 and WT2) were analysed with anti‐HRP. Silver (S.) staining serves as the loading control.

To generate a plant expression vector for down‐regulation of GNTI expression (designated pGNTI‐RNAi), an 882 bp sense strand of the coding sequence of *Nicotiana tabaccum* GNTI and a 508 bp fragment for antisense orientation were cloned into the plant binary vector pGPTV‐HPT (Becker *et al*., 1992) under the control of the cauliflower mosaic virus 35S promoter and terminator (Figure 1a). The *Agrobacterium tumefaciens* carrying pGNTI‐RNAi was used to transform *Nicotiana benthamiana* plants. Five independent hygromycin‐resistant *N. benthamiana*: T_2_ generation plants (herein referred to as *Nb*GNTI‐RNAi plants) were generated. To validate the degree of plant‐specific β1,2‐xylose‐ and/or α1,3‐fucose‐containing *N‐*glycans, total proteins from a *N. benthamiana* wild‐type plant (WT), an *Arabidopsis thaliana complex‐glycan‐deficient* plant (*cgl*) and individual *Nb*GNTI‐RNAi plants were subjected to immunoblot analysis. Protein extracts were separated with SDS‐PAGE, blotted onto PVDF membrane and then detected using an anti‐HRP antibody specific for plant complex‐type *N*‐glycans (Lauriere *et al*., 1989). Binding of the anti‐HRP antibody to several proteins was detected in protein extract from the WT, indicating the presence of proteins carrying β1,2‐xylose and/or α1,3‐fucose epitopes (Figure 1b). On the other hand, the β1,2‐xylose and/or α1,3‐fucose was undetectable in protein extract from the *cgl* plant, as expected (Figure 1b), as the absence of GNTI activity prevented complex‐type *N‐*glycans synthesis (von Schaewen *et al*., 1993; Strasser *et al*., 2005). In the protein extract from *Nb*GNTI‐RNAi7, β1,2‐xylose and/or α1,3‐fucose‐containing epitopes of glycoproteins were significantly, but not completely, reduced compared with other *Nb*GNTI‐RNAi transgenic lines (Figure 1b). These *Nb*GNTI‐RNAi lines did not show any obvious phenotype under standard growth conditions (data not shown). The *Nb*GNTI‐RNAi7 was stable and could be transferred along with the transgene over at least six generations (Figure 1c).

### Silencing of β1,2‐*N*‐acetylglucosaminyltransferase I leads to a marked increase in the Man5GlcNAc2 structure and a marked reduction in the xylosylated and core fucosylated *N*‐glycan structures of glycoproteins

**Figure 2:**
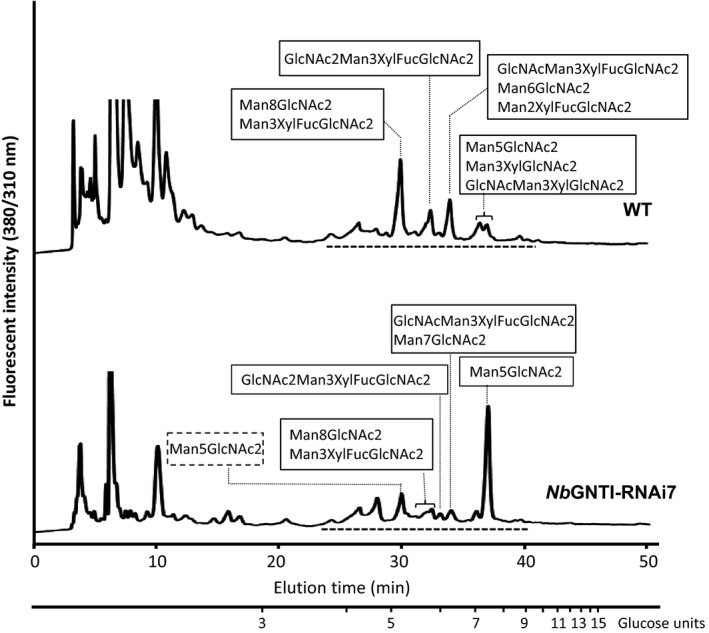
Glycan profiles of WT and *Nb* GNTI‐RNAi7 (T_5_ generation). Total *N*‐glycans from glycoproteins were prepared by hydrazinolysis and labelled with 2‐aminopyridine (PA). PA‐labelled glycans were analysed by RP‐HPLC using a C 18 column. All peaks (indicated by a broken line) were then subjected to LC–MS/MS. The glycan structures identified from deconvoluted MS/MS spectra are indicated in the box. The M5 structure of *Nb* GNTI‐RNAi7 eluted earlier (indicated by a broken line box) are not included in the calculation.

To observe the *N*‐glycan composition changes in *Nb*GNTI‐RNAi7, the total amounts of *N*‐glycans released from glycoproteins of WT and *Nb*GNTI‐RNAi7 were determined by RP‐HPLC and LC–MS/MS. Total *N*‐glycans of WT and *Nb*GNTI‐RNAi7 were liberated by hydrazinolysis and labelled with 2‐aminopyridine (PA). PA‐glycans were analysed by RP‐HPLC using a C18 column, and the collected peaks shown in Figure 2 were further subjected to LC–MS/MS to determine the structure. The glycan structures of WT were M2XF (4.3%), M3X (6.4%), M3XF (48.2%), GNM3X (3.6%), GNM3XF (7.8%), GN2M3XF (19.0%), M5 (4.8%), M6 (2.4%) and M8 (3.5%) (M, mannose; GN, *N*‐acetylglucosamine; F, fucose; X, xylose). In contrast, the *N*‐glycan structures of *Nb*GNTI‐RNAi7 were M3XF (1.2%), GNM3XF (2.7%), GN2M3XF (5.2%), M5 (82.4%), M7 (4.1%) and M8 (4.4%), as shown in Table 1.

**Table 1:** Composition of sugar chain structures of WT and *Nb* GNTI‐RNAi7

Abbreviation	Structure	Relative amount (%)	
		WT	*Nb*GNTI‐RNAi7
M2XF	Man2XylFucGlcNAc2	4.3	–
M3X	Man3XylGlcNAc2	6.4	–
M3XF	Man3XylFucGlcNAc2	48.2	1.2
GNM3X	GlcNAcMan3XylGlcNAc2	3.6	–
GNM3XF	GlcNAcMan3XylFucGlcNAc2	7.8	2.7
GN2M3XF	GlcNAc2Man3XylFucGlcNAc2	19.0	5.2
**Plant‐type structures**		**89.3**	**9.1**
M5	Man5GlcNAc2	4.8	82.4
M6	Man6GlcNAc2	2.4	–
M7	Man7GlcNAc2	–	4.1
M8	Man8GlcNAc2	3.5	4.4
**Mannose‐type structures**		**10.7**	**90.9**

### Successful cross‐pollination of a GNTI‐knockdown plant with a human glucocerebrosidase expressing *N. benthamiana* plant

**Figure 3:**
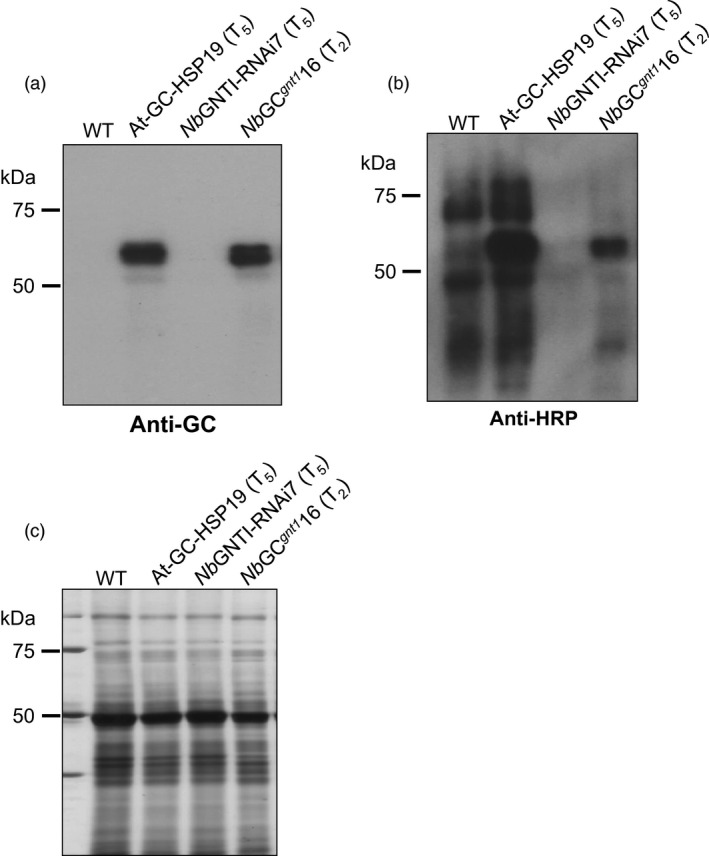
T_2_ generation of cross‐pollinated *Nb* GNTI‐RNAi and At‐GC‐HSP19 (*Nb* GC ^*gnt1*^) *Nicotiana benthamiana* plants. Total soluble protein (1 μg) was loaded in each lane on 10% SDS‐PAGE gel and then analysed by immunoblotting using (a) anti‐GC antibody or (b) anti‐HRP antibody. (c) Silver staining serves as the loading control.

To generate human glucocerebrosidase (GC) in glyco‐engineered *N. benthamiana* plants, a cross‐pollination between a GNTI down‐regulated plant (*Nb*GNTI‐RNAi7) and GC‐expressing plant (At‐GC‐HSP19) was performed. A total of 30 hygromycin/bialaphos‐resistant *N. benthamiana* T_1_ generation plants (designated *Nb*GC^*gnt1*^ plants) were generated. Each *Nb*GC^*gnt1*^ plant was subjected to immunoblot analysis and GC activity assay. The GC gene was successfully cross‐pollinated to the *Nb*GNTI‐RNAi7 plant (Figure S1). The *Nb*GC^*gnt1*^16 was selected because it showed the highest GC activity (Figure S1c), and then, its T_2_ generation was generated. Protein extracts from the WT, At‐GC‐HSP19 and *Nb*GNTI‐RNAi7 plants, and T_2_ generation from the *Nb*GC^*gnt1*^16 plant were visualized by immunoblotting using anti‐HRP antibody. The same membrane was stripped and reblotted with anti‐GC antibody. The visualized GC band of *Nb*GC^*gnt1*^16 indicated that the GC gene could be passed along to the next generation by self‐pollination (Figure 3a). The WT and At‐GC‐HSP19 proteins, carrying β1,2‐xylose and/or α1,3‐fucose epitopes, had strong binding signals to anti‐HRP antibody, whereas the protein extracts from *Nb*GNTI‐RNAi7 did not exhibit detectable binding signals (Figure 3b). The equivalence in the amounts of loaded protein was confirmed with silver staining (Figure 3c). The anti‐HRP antibody binding against proteins from *Nb*GC^*gnt1*^16 was strongly reduced in comparison with At‐GC‐HSP19, but the signals were increased compared to *Nb*GNTI‐RNAi7, revealing that the plant‐specific *N*‐glycans were dramatically reduced. However, the RP‐HPLC chromatogram of PA‐labelled glycans from *Nb*GC^*gnt1*^16 had a pattern similar to *Nb*GNTI‐RNAi7 (Figure S2).

### Purified GC derived from GNTI‐knockdown *N. benthamiana* plants has a predominantly high‐mannose‐type *N*‐glycan structure

**Figure 4:**
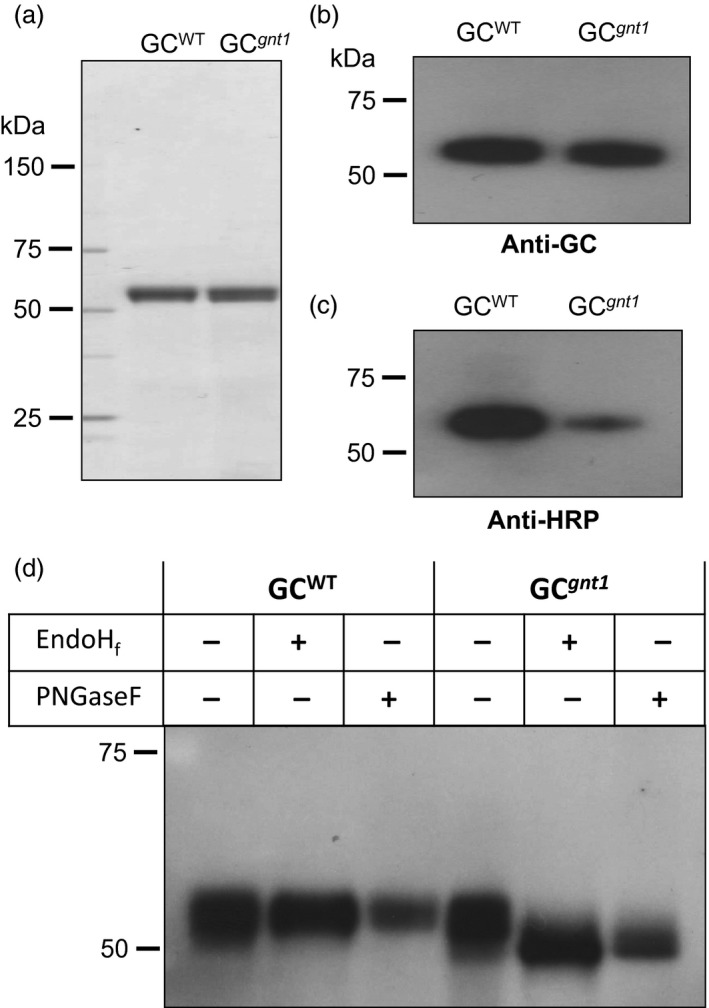
Glycan characterization of purified GC produced in WT (GC ^WT^) and GNTI suppression (GC ^*gnt1*^) *Nicotiana benthamiana* plants. The purified GC ^WT^ and GC ^*gnt1*^ were analysed on 5%–20% SDS‐PAGE gel and (a) stained with Coomassie Brilliant Blue (1 μg), and also analysed by immunoblot (100 ng) using (b) anti‐GC antibody or (c) anti‐HRP antibody. (a–b) Equal amounts of purified GC ^WT^ and GC ^*gnt1*^ were analysed while (c) the purified GC ^*gnt1*^ showed a smaller amount of plant complex *N*‐glycans compared with GC ^WT^. (d) One hundred ng of each sample was digested in the absence (−) or presence (+) of either EndoH_f_ or PNGase F and analysed on 7.5% SDS‐PAGE gel. The GC bands were visualized by immunoblotting.

To characterize the WT‐derived GC from At‐GC‐HSP19 (designated GC^WT^) and GNTI‐knockdown‐derived GC from *Nb*GC^*gnt1*^16 (designated GC^*gnt1*^), the GC was purified by two‐steps using concanavalin A (Con A) chromatography and Phenyl 650C chromatography. The yield of GC^WT^ and GC^*gnt1*^ was 10.6% and 11.1%, respectively (Table S2). By SDS‐PAGE, the purified GC^WT^ and GC^*gnt1*^ showed a single band indicating the purity of purified GC^WT^ and GC^*gnt1*^ (Figure 4a). The immunoblot analysis and deglycosylation were used to further characterize the difference of dominant *N*‐glycans between purified GC^WT^ and GC^*gnt1*^. When the concentration of purified GC^WT^ and GC^*gnt1*^ were equal as visualized by anti‐GC antibody (Figure 4b), the purified GC^*gnt1*^ showed a smaller amount of plant complex *N*‐glycans against anti‐HRP antibody compared with GC^WT^ (Figure 4d). To release the carbohydrate moieties, purified GC^WT^ and GC^*gnt1*^ were treated with EndoH_f_, which is able to remove a high‐mannose‐type *N*‐glycan, or with peptide‐*N*‐glycosidase (PNGase) F, which is unable to remove the complex α1,3‐fucosylated glycans of plant glycoproteins (Tretter *et al*., 1991). The GC^*gnt1*^ treated with EndoH_f_ showed a band shift, revealing that the high‐mannose‐type structure of GC^*gnt1*^ was removed. In contrast, the GC^WT^ treated with EndoH_f_ did not show the difference in size (Figure 4d). The shifted band of GC^*gnt1*^ treated with PNGaseF indicated that the GC^*gnt1*^ mainly contained a non‐α1,3‐fucosylated plant glycan. Unlike in the case of GC^WT^, the glycan structure contained α1,3‐fucosylated glycan that could not be cleaved by PNGaseF (Figure 4d).

### All four of the occupied *N*‐glycosylation sites of GC^*gnt1*^ have a predominantly Man5GlcNAc2 structure

**Figure 5:**
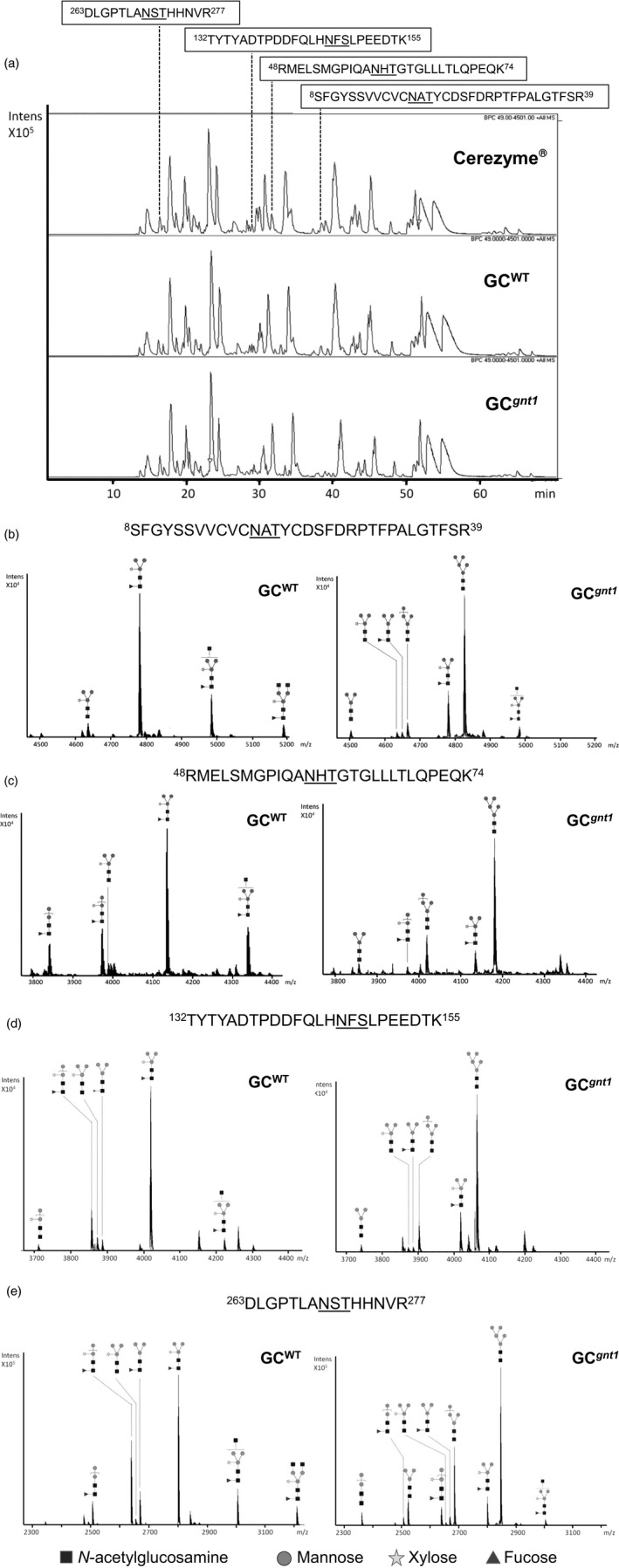
Nano LC–MS spectra of tryptic glycopeptides derived from purified GC ^WT^ and GC ^*gnt1*^. The purified GC ^WT^ and GC ^*gnt1*^ were excised from a gel, trypsinized and then subjected to nano LC–MS/MS. (a) The elution pattern of tryptic peptide derived from Cerezyme^®^, GC ^WT^ and GC ^*gnt1*^ on nano HPLC. The glycan structures were identified from deconvoluted MS/MS spectra. (b–e) show the glycoforms of glycopeptides with *N*‐glycosylation sites N19, N59, N146 and N270, respectively.

To identify constituent *N*‐glycans of each *N*‐glycosylation site, the tryptic GC^WT^ and GC^*gnt1*^ peptides were analysed by *de novo* sequencing using nanoLC‐MS/MS analysis. The GC contains five potential *N*‐glycosylation sites (Asn‐X‐Ser/Thr) but only the first four sites are generally occupied in all natural GC (Takasaki *et al*., 1984) and commercial GC produced in CHO cells, fibroblast cells (Brumshtein *et al*., 2010) and carrot suspension cells (Shaaltiel *et al*., 2007). The peptide fragments containing the last potential *N*‐glycosylation site (Asn462) of both GC^WT^ and GC^*gnt1*^ were detected at 2305 m/z indicating the unoccupied Asn462 site. The same fragment size was also detected in Cerezyme^®^ (Figure S3). The tryptic peptides of Cerezyme^®^, GC^WT^ and GC^*gnt1*^ showed similar pattern on nano HPLC (Figure 5a). In addition, the extracted‐ion chromatogram (EIC) indicated that glycopeptides bearing the sequence of Asn19 (^8^SFGYSSVVCVCNATYCDSFDRPTFPALGTFSR^39^), Asn59 (^48^RMELSMGPIQANHTGTGLLLTLQPEQK^74^), Asn146 (^132^TYTYADTPDDFQLHNFSLPEEDTK^155^) and Asn270 (^263^DLGPTLANSTHHNVR^277^) were eluted at about 38, 33, 30 and 16 min, respectively (Figure 5a). These *N*‐glycosylation sites of GC^WT^ and GC^*gnt1*^ contained predominantly the M3XF structure and M5 structure (Figure 5b–e). The *N*‐glycan structures of GC^WT^ contained 100% plant‐type structures with β1,2‐xylose and/or α1,3‐fucose epitopes in all of the *N*‐glycosylation sites (Table 2). In contrast, the *N*‐glycan structures of GC^*gnt1*^ mostly contained mannose‐type structures which ranged from 73.3% up to 85.5% of the total structures (Table 2). The original human signal peptide of GC could function properly and was removed correctly in *N. benthamiana*, and thus the *N*‐terminal tryptic peptide sequence, ^1^ARPCIPK^7^, was detected in both GC^WT^ and GC^*gnt1*^ just as in Cerezyme^®^ (Figure S3). The *N*‐glycan structures of Cerezyme^®^ were also analysed and revealed that the structural profile was consistent with a previous report by Brumshtein *et al*. (2010) that the Asn19 site mostly carried an Man3GlcNAc2 structure and the other sites contained a chitobiose tri‐mannosyl core glycan with fucosylation (Figure S4).

**Table 2:** Composition of sugar chain structures attached on GC ^WT^ and GC ^*gnt1*^

Abbreviation	Structure	N19 (S8‐R39)		N59 (R48‐K74)		N146 (T132‐K155)		N270 (D263‐R277)	
		GC^WT^	GC^*gnt1*^	GC^WT^	GC^*gnt1*^	GC^WT^	GC^*gnt1*^	GC^WT^	GC^*gnt1*^
		Relative amount (%)							
M2X	Man2XylGlcNAc2	–	–	–	–	2.7	–	–	–
M2F	Man2FucGlcNAc2	5.7	–	–	–	–	–	6.7	2.7
M2XF	Man2XylFucGlcNAc2	20.0	–	11.3	3.4	16.5	–	23.9	0.7
M3X	Man3XylGlcNAc2	6.3	2.5	1.8	–	5.5	2.3	1.5	0.9
M3F	Man3FucGlcNAc2	–	2.5	4.0	–	4.6	2.5	9.2	2.7
M3XF	Man3XylFucGlcNAc2	68.1	20.6	56.1	11.1	65.5	18.6	43.5	9.1
GNM3XF	GlcNAcMan3XylFucGlcNAc2	–	1.1	19.5	–	5.2	–	10.0	1.9
GN2M3XF	GlcNAc2Man3XylFucGlcNAc2	–	–	7.2	–	–	–	5.2	–
**Plant‐type structures**		**100**	**26.7**	**100**	**14.5**	**100**	**23.4**	**100**	**18.0**
M2	Man2GlcNAc2	–	–	–	–	–	–	–	1.3
M3	Man3GlcNAc2	–	3.2	–	5.1	–	–	–	8.3
M4	Man4GlcNAc2	–	6.9	–	18.0	–	3.5	–	23.9
M5	Man5GlcNAc2	–	63.2	–	62.5	–	73.0	–	48.6
**Mannose‐type structures**		**–**	**73.3**	**–**	**85.5**	**–**	**76.6**	**–**	**82.0**

### GC^WT^ and GC^*gnt1*^ are taken up by macrophages via mannose receptors

**Figure 6:**
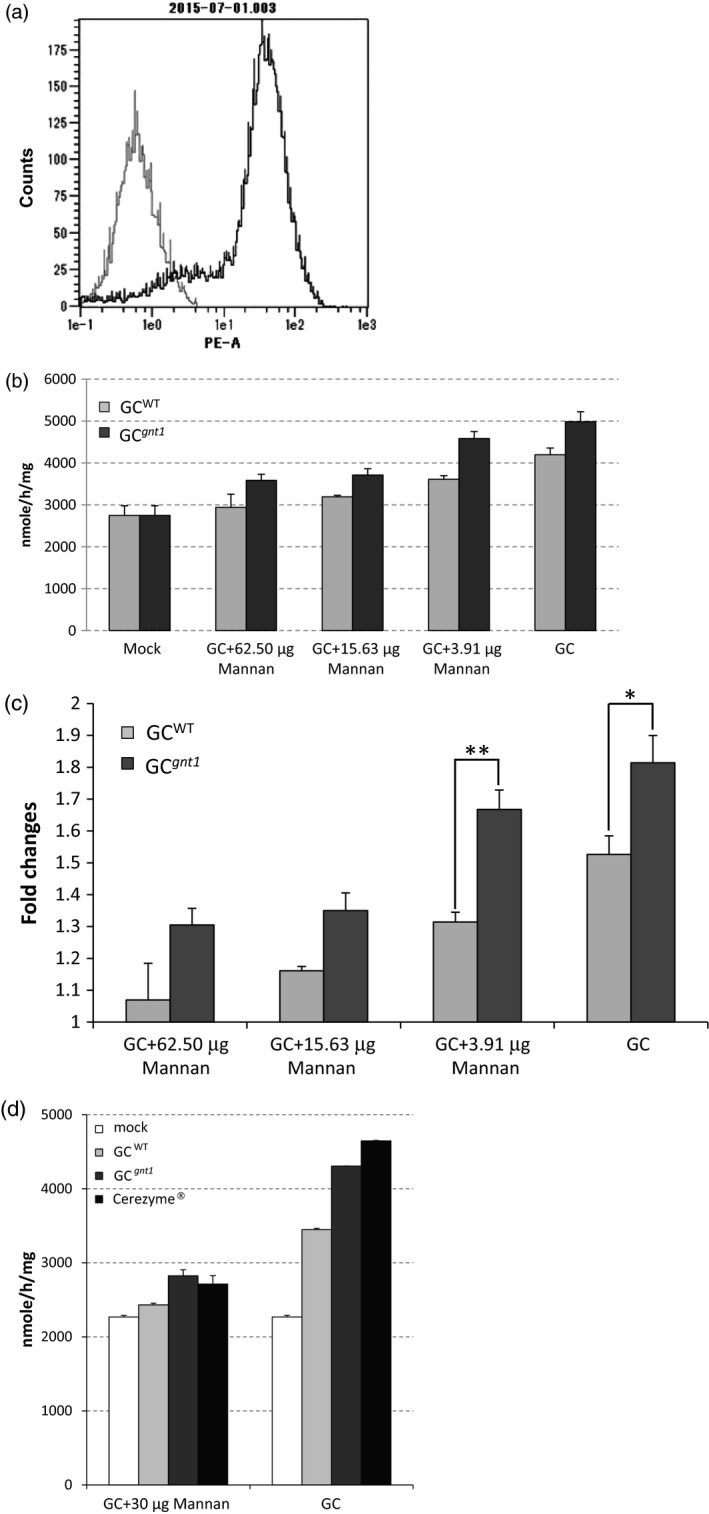
Uptake of GC ^WT^ or GC ^*gnt1*^ by macrophages via mannose receptors. The macrophage cells were stained with the macrophage marker CD11b. Unstained cells (negative control, grey) and macrophages (black) were analysed by flow cytometry (a). The effect of mannan on the specific uptake of GC ^WT^ and GC ^*gnt1*^ was determined (b), and the cellular activities of GC ^WT^ and GC ^*gnt1*^ were compared (c). The cellular activities of GC ^WT^, GC ^*gnt1*^ and Cerezyme^®^ were compared (d). The results and error bars represent the mean ± SE (*n* = 3), significantly different at the level of **P < 0.05; *P = 0.05.

Macrophage cells prepared from C57BL/6J mice were used to determine the targeting and uptake of GC^WT^ and GC^*gnt1*^ by macrophages. The purity of macrophage cells was 99.4% as determined by flow cytometry (Figure 6a). The uptake of GC^WT^ and GC^*gnt1*^ was inhibited by mannan in a dose‐dependent manner, confirming that GC^WT^ and GC^*gnt1*^ were taken up into macrophage cells via mannose receptors (Figure 6b). The intracellular activity of GC^*gnt1*^ with 3.91 μg mannan was significantly higher than the activity of GC^WT^ (*P *= 0.011). Under the condition without mannan, the intracellular activities of GC^WT^ and GC^*gnt1*^ were increased 1.5‐ and 1.8‐fold, respectively, relative to untreated cells. Therefore, the cellular activity of GC^*gnt1*^ was higher than that of GC^WT^, and the difference was nearly significant (*P *= 0.050; Figure 6c). Comparing with Cerezyme^®^ under the condition without mannan, the intracellular activities of Cerezyme^®^ showed significantly higher than that of GC^WT^ (*P *= 0.014) and GC^*gnt1*^ (*P *= 0.018; Figure 6d). The intracellular activity of GC^*gnt1*^ without mannan was significantly higher than the activity of GC^WT^ (*P *= 0.018). In the present of mannan, the intracellular activities of mock, GC^WT^, GC^*gnt1*^ and Cerezyme^®^ were not significantly difference from each others.

### GC^WT^ and GC^*gnt1*^ are distributed into the liver and spleen

**Figure 7:**
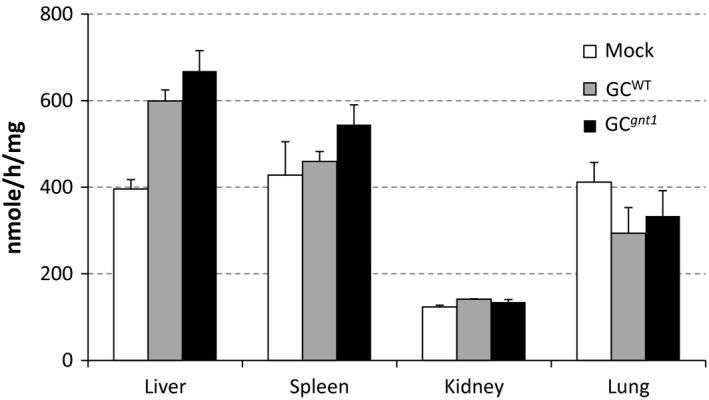
Uptake of GC ^WT^ or GC ^*gnt1*^ into the organs of C57BL/6J mice. The distribution to the liver, spleen, kidney and lungs of mice was evaluated by enzymatic activity at 60 min postinjection. The results and error bars represent the mean ± SE (*n* = 3 mice).

The distribution of GC^WT^ and GC^*gnt1*^ was assessed in C57BL/6J mice by measuring enzyme activity in the liver, spleen, kidney and lung at 60 min after single injection. The GC^WT^ and GC^*gnt1*^ were taken up into the liver and spleen, the primary target organs for the treatment of Gaucher's disease, in contrast with the kidney and lung (Figure 7). No significant differences were found between the uptake of the two enzymes in either the liver (*P *= 0.267) or spleen (*P *= 0.171). Comparing with PBS‐injected mice (mock), GC^WT^ and GC^*gnt1*^ showed significantly increase in the liver but did not showed significantly increase in the spleen.

## Discussion

Plants are a promising alternative for biopharmaceutical production; however, the potential immunogenicity of plant‐specific *N*‐glycan structures continues to be a concern (Bencurova *et al*., 2004; van Ree *et al*., 2000; Tretter *et al*., 1993). Various efforts have been made to produce human‐like or customized *N*‐glycan structures in plants to enable the use of plants as a safe and efficient biopharmaceutical production system. Here we reported the first successful generation of transgenic down‐regulated GNTI *N. benthamiana* plants, which drastically reduced plant‐specific carbohydrate epitopes. The plant‐specific *N*‐glycan structures accounted for only 9.1% of the total *N*‐glycan structures in *Nb*GNTI‐RNAi7, while in the WT plants the plant‐specific glycans made up as much as 89.3% of the total *N*‐glycan structures (Table 1). This *Nb*GNTI‐RNAi7 mutant could be used as a host for biopharmaceutical productions. For instance, using these transgenic plants with a magniCON^®^ transient expression system might be able to achieve up to 80% TSP (Marillonnet *et al*., 2005). The magniCON^®^ system was reported to generate as much as 1–2 mg/g of fresh weight for malarial antigen production within a week postinfection (Webster *et al*., 2009). Interestingly, this mutant, as the Arabidopsis *cgl* mutant, did not display any severe phenotype under the normal growing conditions. In contrast to Arabidopsis *cgl*,* GNTI*‐knockout in *Oryza sativa* showed severe phenotypes with arrested seedling development and lethality before reaching the reproductive stage (Fanata *et al*., 2013). A possible explanation may be that complex *N*‐glycans (or individual sugar residue) are essential for the growth and development in some plant species such as rice (Strasser, 2014). A previous study on the down‐regulation of the endogenous *XylT* and *FucT* genes in *N. benthamiana* showed that these plants were also viable and did not show an obvious phenotype under laboratory conditions (Strasser *et al*., 2008). In addition, the knockout of two *XylT* and two *FucT* genes in *N. benthamiana* plants using transcription activator‐like effector nucleases (TALENs) was able to produce seeds and generate next‐generation plants (Li *et al*., 2015). Therefore, the complex *N*‐glycans might not be as crucial for *N. benthamiana* as for *A. thaliana*. Another explanation would be that a small amount of complex *N*‐glycans (about 9.1% in this study) may be sufficient for the viability of *N. benthamiana*. To investigate this possibility, a *gnt1* knockout mutant of *N. benthamiana* such as that previously reported using a CRISPR/Cas9 system (Ran *et al*., 2013) should be further investigated.

The CHO‐derived GC for Gaucher's disease is one of the most expensive drugs in the world. The treatment costs about 200 000 US dollars per year, as reviewed by Wood *et al*. (2013). Therefore, there is a pressing need for a system for low‐cost mass production GC at a reasonable price. In this study, we generated a *Nb*GC^*gnt1*^ transgenic line that could produce GC with the desired *N*‐glycans, predominantly with the Man5GlcNAc2 structure. However, purified GC^*gnt1*^ still exhibited some xylose and/or fucose moieties (ranging from 14.5% to 26.7%) on the *N*‐glycans that might have been due to an incomplete GNTI silencing. On the other hand, the *N*‐glycan status of recombinant glycoproteins appears to be protein‐specific as shown in the GC produced in seeds of the Arabidopsis *cgl* mutant (Kermode, 2012). Plant‐specific *N*‐glycan was undetectable among the total glycoproteins of this mutant (Strasser *et al*., 2005), whereas Arabidopsis *cgl*‐derived GC contained as much as 15% plant‐specific *N*‐glycan structures (He *et al*., 2012) but only 5.5% of plant‐specific *N*‐glycans were detected in α‐l‐iduronidase produced in the seeds of the same mutant (He *et al*., 2013). If necessary, these plant‐specific *N*‐glycans of GC^*gnt1*^ can be completely removed using an anti‐HRP affinity column as reported by He *et al*. (2012). Interestingly, our immunoblotting analysis revealed that the level of plant‐specific *N*‐glycan was higher in the *Nb*GC^*gnt1*^16 plant than the *Nb*GNTI‐RNAi7 plant (Figure 3b). However, the total *N*‐glycan profiles from both plants were not significantly changed (the plant‐type *N*‐glycan of *Nb*GNTI‐RNAi7 was 9.1% and that of *Nb*GC^*gnt1*^16 was 11.3%), as shown in Figure S2 and Table S1.

The terminal mannose residues are vital for targeting and uptake of GC via macrophage mannose receptors. In the case of Cerezyme^®^, CHO‐derived GC is produced as a mammalian complex type and then *in vitro* deglycosylated with α‐neuraminidase, β‐galactosidase and β‐*N*‐acetylglucosaminidase for exposure of the terminal mannose residues (Friedman and Hayes, 1996; Grace and Grabowski, 1990). For Elelyso^®^, carrot‐derived GC was successfully produced with terminal mannose using a vacuole‐targeting signal. It has been considered that the *trans*‐Golgi trimming of terminal *N*‐acetylglucosamine residues occurs in the vacuole (Vitale and Chrispeels, 1984) by the function of vacuolar β‐*N*‐acetylhexosaminidase (HEXO1; Liebminger *et al*., 2011), resulting in accumulation of the Man3XylFucGlcNAc2 (M3XF) structure of vacuolar glycoproteins. Our finding revealed that a native human signal peptide of GC could properly target GC into the ER in wild‐type *N. benthamiana*. GC was then passed through the *trans*‐Golgi before being directed to the vacuole or secreted into the apoplast. Moreover, the same predominant M3XF structure was presented in *N. benthamiana*‐derived GC as found in Elelyso^®^ by the action of HEXO1 or plasma membrane β‐*N*‐acetylhexosaminidases (HEXO2/3; Strasser *et al*., 2007). However, the Elelyso^®^ has two additional residues at the N‐terminus (EF), derived from the linker used for the fusion of the signal peptide, and seven additional residues at the C‐terminus (DLLVDTM), derived from the encoding sequences of the vacuole‐targeting signal from tobacco chitinase A (Shaaltiel *et al*., 2007). The human GC gene used in this study derived from human liver cDNA did not have a mutation, unlike the Elelyso^®^ and Cerezyme^®^ that have a R495H mutation. It contained original 39 amino acid signal peptide, but the GC gene coding for the production of Cerezyme^®^ has a shorter coding sequence of signal peptide (19 amino acid). However, our results revealed that the human signal peptide in GC^WT^ and GC^*gnt1*^ was probably removed in the same manner as in human and in CHO cells, resulting in detection of the *N*‐terminal tryptic peptide sequence (^1^ARPCIPK^7^) just as in Cerezyme^®^ (Figure S3). The study of He *et al*. (2012) showed that most of the GC expressed without a vacuole‐targeting signal was secreted and predominantly presented within the extracellular spaces in the seeds of the Arabidopsis *cgl* mutant. Therefore, it is highly possible that the majority of GC expressed in both the WT and *GNTI*‐knockdown *N. benthamiana* in this study was also secreted into the apoplast via the default pathway. The removal of the terminal *N*‐acetylglucosamine residues revealed the predominant M3XF structure of GC^WT^ may resulted from the function of HEXO2/3 on plasma membrane (Strasser *et al*., 2007). These data are correlated with the previous study that *cgl*‐derived GC, localized in apoplast, contained higher amount of M3XF (9.2%) than the amount of GNM3XF (2.7%) and GN2M3XF (not detected; He *et al*., 2012).

The differences in glycosylation of GC^WT^ (M3XF) and GC^*gnt1*^ (M5) may affect the targeting to and internalization by macrophages *in vitro* and also the biodistribution and uptake into organs *in vivo*. GC^*gnt1*^ provided a statistically significant improvement in cellular activity both under the condition with the lowest amount of mannan and in the condition without mannan (*P *≤ 0.05). It is possible that the higher amount of terminal *N*‐acetylglucosamine structures of GC^WT^ that did not contribute to the uptake resulting in the lower cellular activity compared with that of GC^*gnt1*^. However, both GC^WT^ and GC^*gnt1*^ had lower cellular activity compared with Cerezyme^®^. An *in vivo* study in WT mice demonstrated that GC^WT^ and GC^*gnt1*^ were distributed and taken up into the liver and spleen but were not taken up into the kidney and lung. This result is not unexpected, because the liver and spleen are the primary target organs for Gaucher's disease. However, the biodistribution of GC^WT^ and GC^*gnt1*^ to spleen is not significantly different compared with the mock because of the high and various background activities in the spleen. GC^*gnt1*^‐injected mice showed a trend towards higher uptake to target organs compared to GC^WT^‐injected mice, but there were no significant differences (*P *> 0.05). The previous studies reported that the longer mannose structures do not seem to affect enzyme uptake into macrophages or uptake to organs in a mouse model of Gaucher's disease (van Patten *et al*., 2007; Tekoah *et al*., 2013). In this study, both *in vitro* and *in vivo* data were obtained from WT mice that exhibited endogenous glucocerebrosidase activities. These background activities might vary among rodents, tissues and cells, which could lead to different data depending on the experimental systems. The therapeutic efficacy using Gaucher's disease in comparison with commercial glucocerebrosidase should be further investigated.

## Supporting information


**Figure S1** T_1_ generation of cross‐pollinated *Nb*GNTI‐RNAi7 and At‐GC‐HSP19 *N. benthamiana* plants (*Nb*GC^*gnt1*^).
**Figure S2** Glycan profiles of *Nb*GNTI‐RNAi7 (T_5_ generation) and *Nb*GC^*gnt1*^16 (T_2_ generation).
**Figure S3** *De novo* sequencing by Biotools software.
**Figure S4** Nano LC–MS spectra of tryptic glycopeptides of commercial GC (Cerezyme^®^).
**Table S1** Composition of sugar chain structures of *Nb*GNTI‐RNAi7 and *Nb*GC^*gnt1*^16
**Table S2** Purification of GC^WT^ and GC^*gnt1*^
