# Supplementary material for: The production of human glucocerebrosidase in glyco‐engineered Nicotiana benthamiana plants
Source: Plant Biotechnol J. 2016 Feb 12;14(8):1682–94. doi: 10.1111/pbi.12529 (PMC5067671; doi:10.1111/pbi.12529)
Supplement: Supplementary file 1 — Figure S1 T1 generation of cross‐pollinated NbGNTI‐RNAi7 and At‐GC‐HSP19 N. benthamiana plants (NbGC gnt1 ). Figure S2 Glycan profiles of NbGNTI‐RNAi7 (T5 generation) and NbGC gnt1 16 (T2 generation). Figure S3 De novo sequencing by Biotools software. Figure S4 Nano LC–MS spectra of tryptic glycopeptides of commercial GC (Cerezyme®). Table S1 Composition of sugar chain structures of NbGNTI‐RNAi7 and NbGC gnt1 16 Table S2 Purification of GCWT and GC gnt1 [file PBI-14-1682-s001.ppt]

## Slide 1
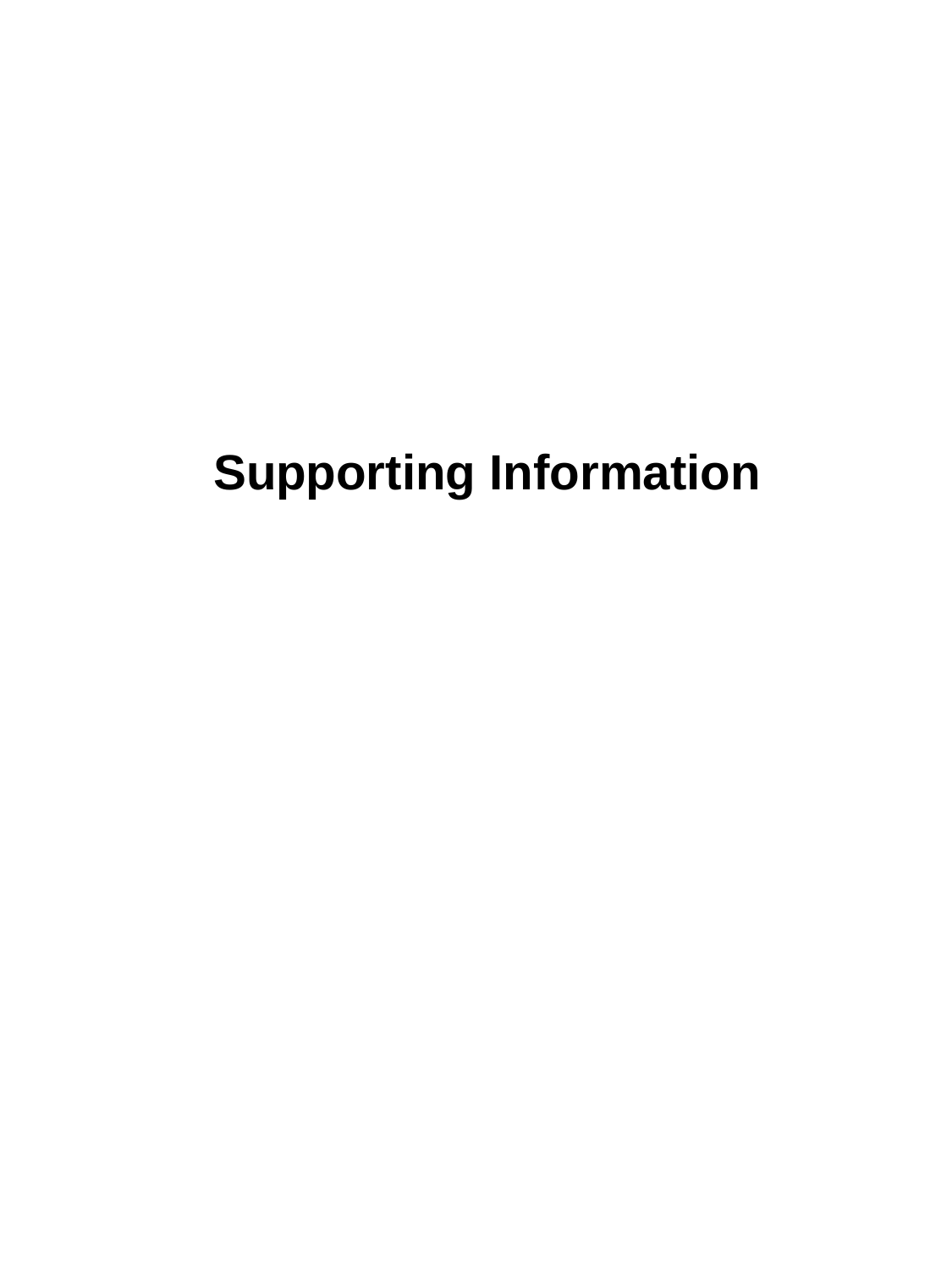

Supporting Information

## Slide 2
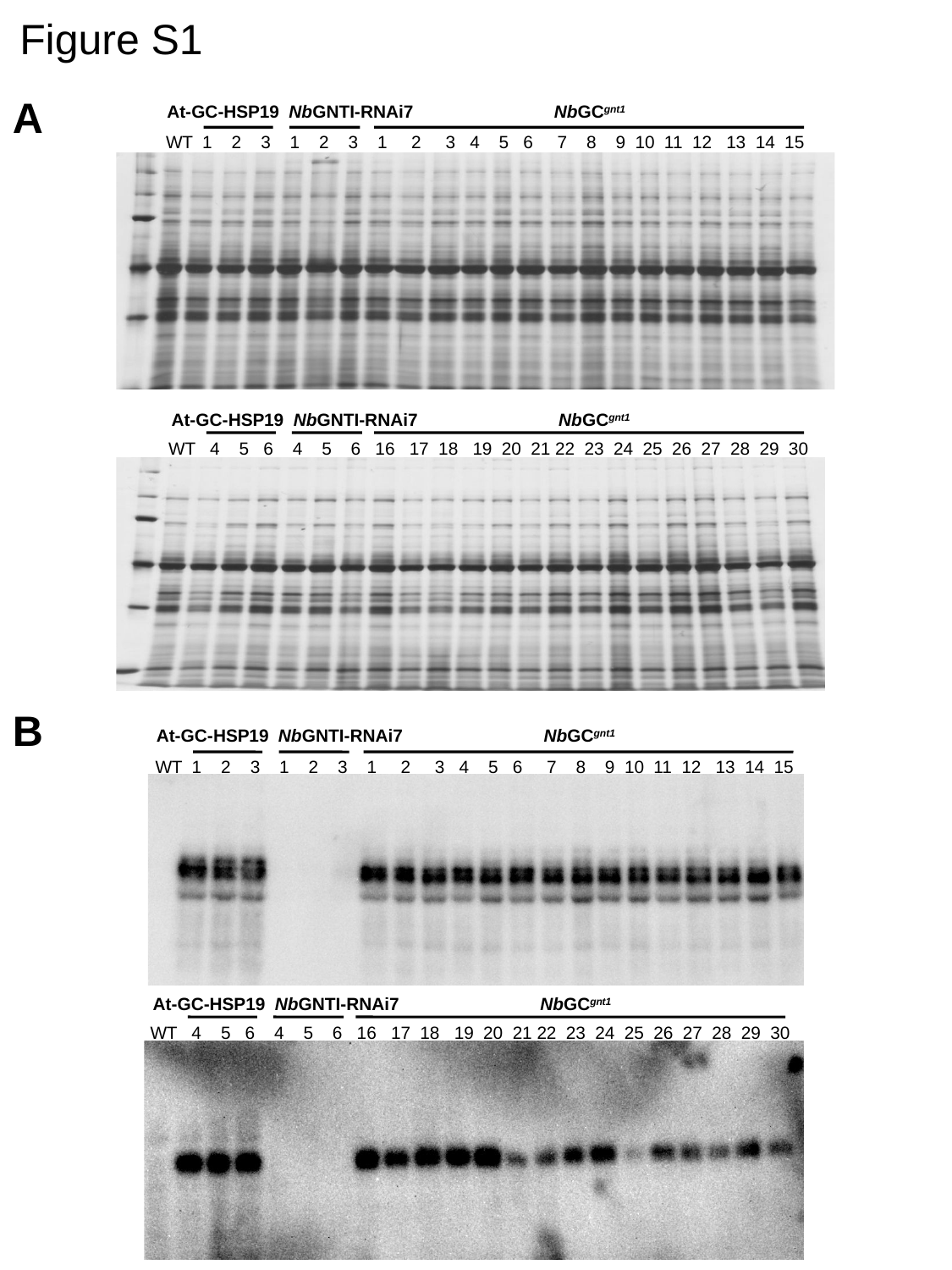

Figure S1
A
 At-GC-HSP19 NbGNTI-RNAi7 NbGCgnt1
 WT 1 2 3 1 2 3 1 2 3 4 5 6 7 8 9 10 11 12 13 14 15
 At-GC-HSP19 NbGNTI-RNAi7 NbGCgnt1
 WT 4 5 6 4 5 6 16 17 18 19 20 21 22 23 24 25 26 27 28 29 30
B
 At-GC-HSP19 NbGNTI-RNAi7 NbGCgnt1
 WT 1 2 3 1 2 3 1 2 3 4 5 6 7 8 9 10 11 12 13 14 15
 At-GC-HSP19 NbGNTI-RNAi7 NbGCgnt1
 WT 4 5 6 4 5 6 16 17 18 19 20 21 22 23 24 25 26 27 28 29 30

## Slide 3
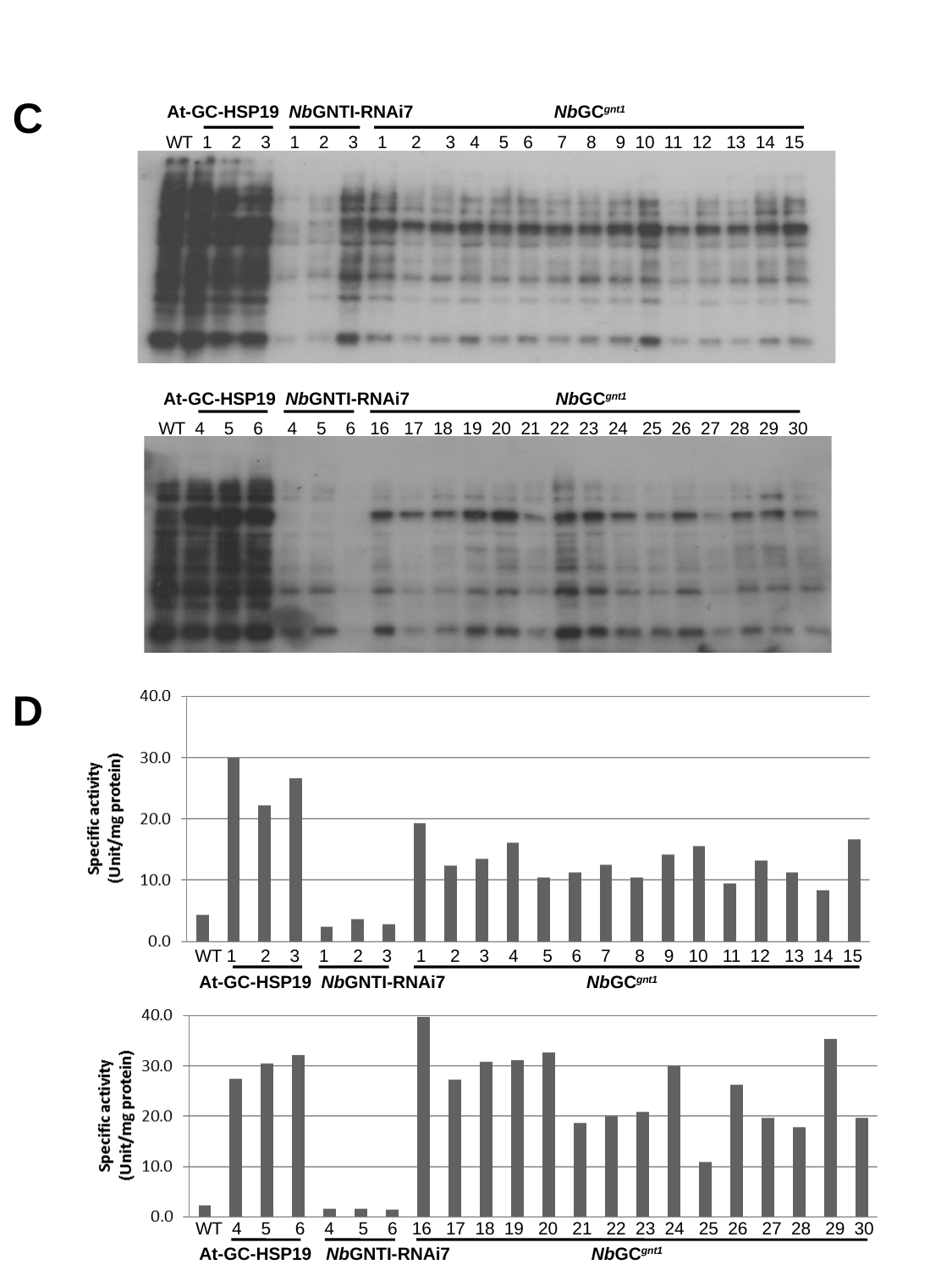

C
 At-GC-HSP19 NbGNTI-RNAi7 NbGCgnt1
 WT 1 2 3 1 2 3 1 2 3 4 5 6 7 8 9 10 11 12 13 14 15
 At-GC-HSP19 NbGNTI-RNAi7 NbGCgnt1
 WT 4 5 6 4 5 6 16 17 18 19 20 21 22 23 24 25 26 27 28 29 30
D
 WT 1 2 3 1 2 3 1 2 3 4 5 6 7 8 9 10 11 12 13 14 15
 At-GC-HSP19 NbGNTI-RNAi7 NbGCgnt1
 WT 4 5 6 4 5 6 16 17 18 19 20 21 22 23 24 25 26 27 28 29 30
 At-GC-HSP19 NbGNTI-RNAi7 NbGCgnt1

## Slide 4
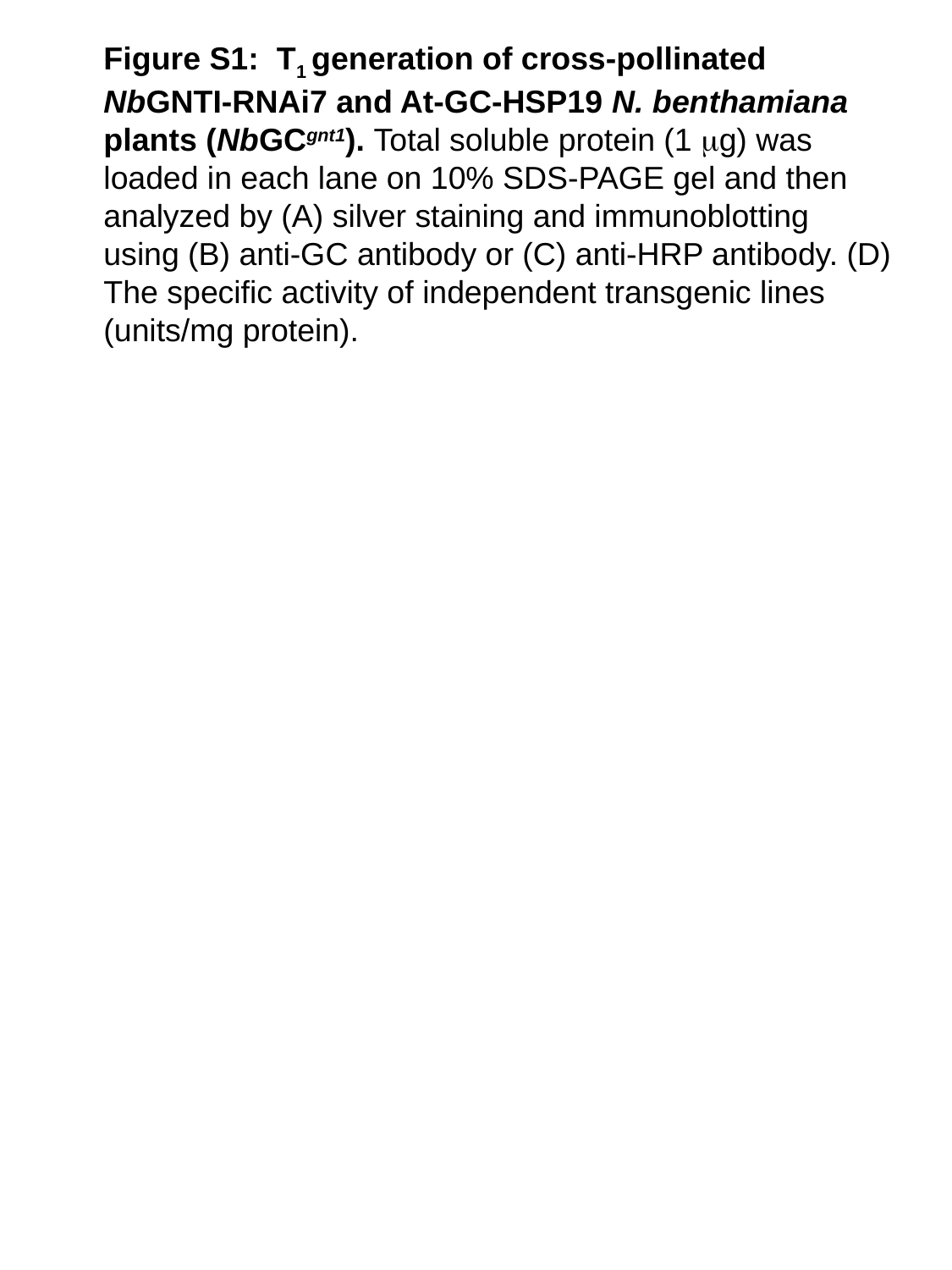

Figure S1: T1 generation of cross-pollinated NbGNTI-RNAi7 and At-GC-HSP19 N. benthamiana plants (NbGCgnt1). Total soluble protein (1 μg) was loaded in each lane on 10% SDS-PAGE gel and then analyzed by (A) silver staining and immunoblotting using (B) anti-GC antibody or (C) anti-HRP antibody. (D) The specific activity of independent transgenic lines (units/mg protein).

## Slide 5
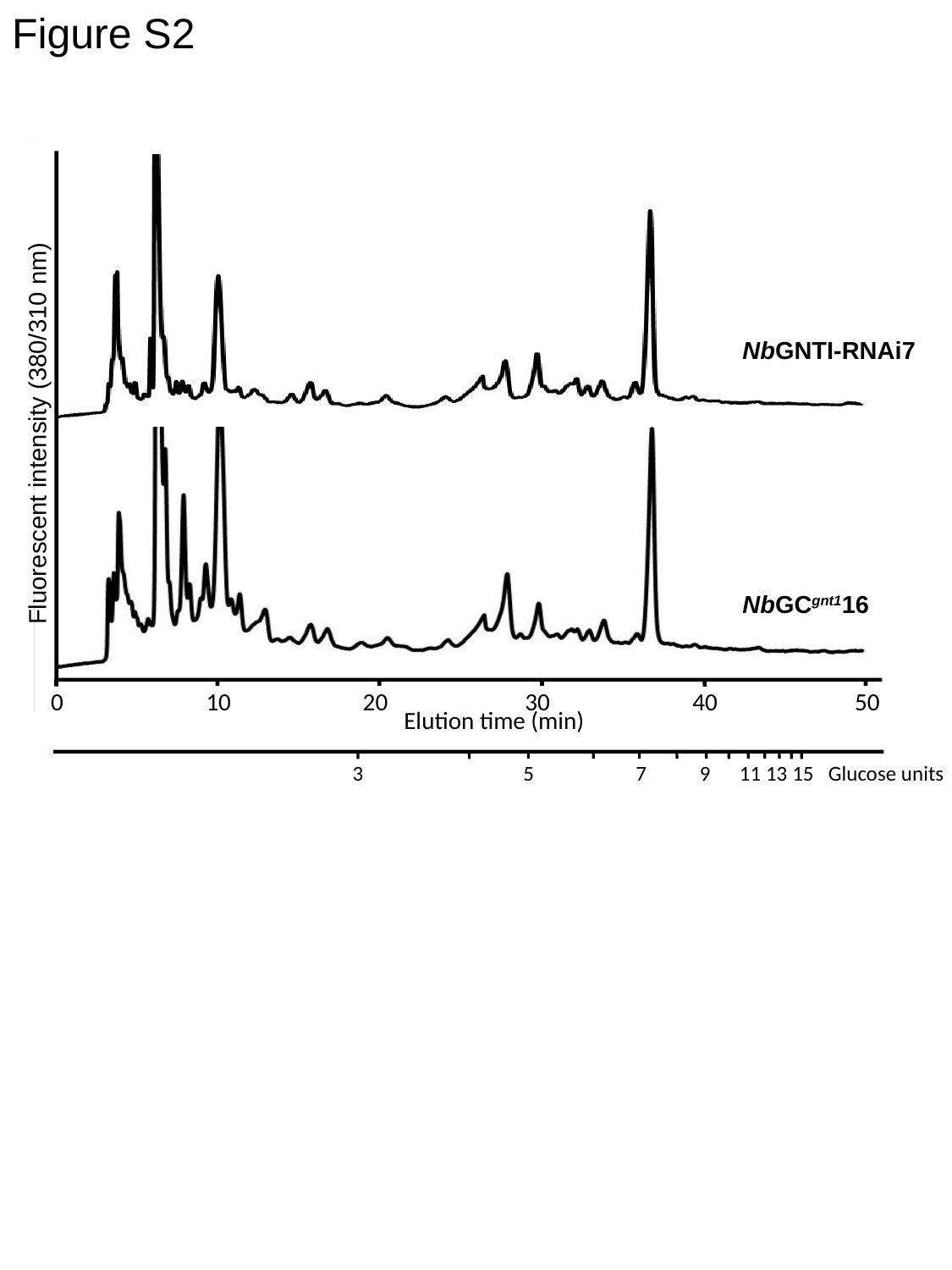

Figure S2
NbGNTI-RNAi7
Fluorescent intensity (380/310 nm)
NbGCgnt116
0 10 20 30 40 50
Elution time (min)
 3 5 7 9 11 13 15 Glucose units

## Slide 6
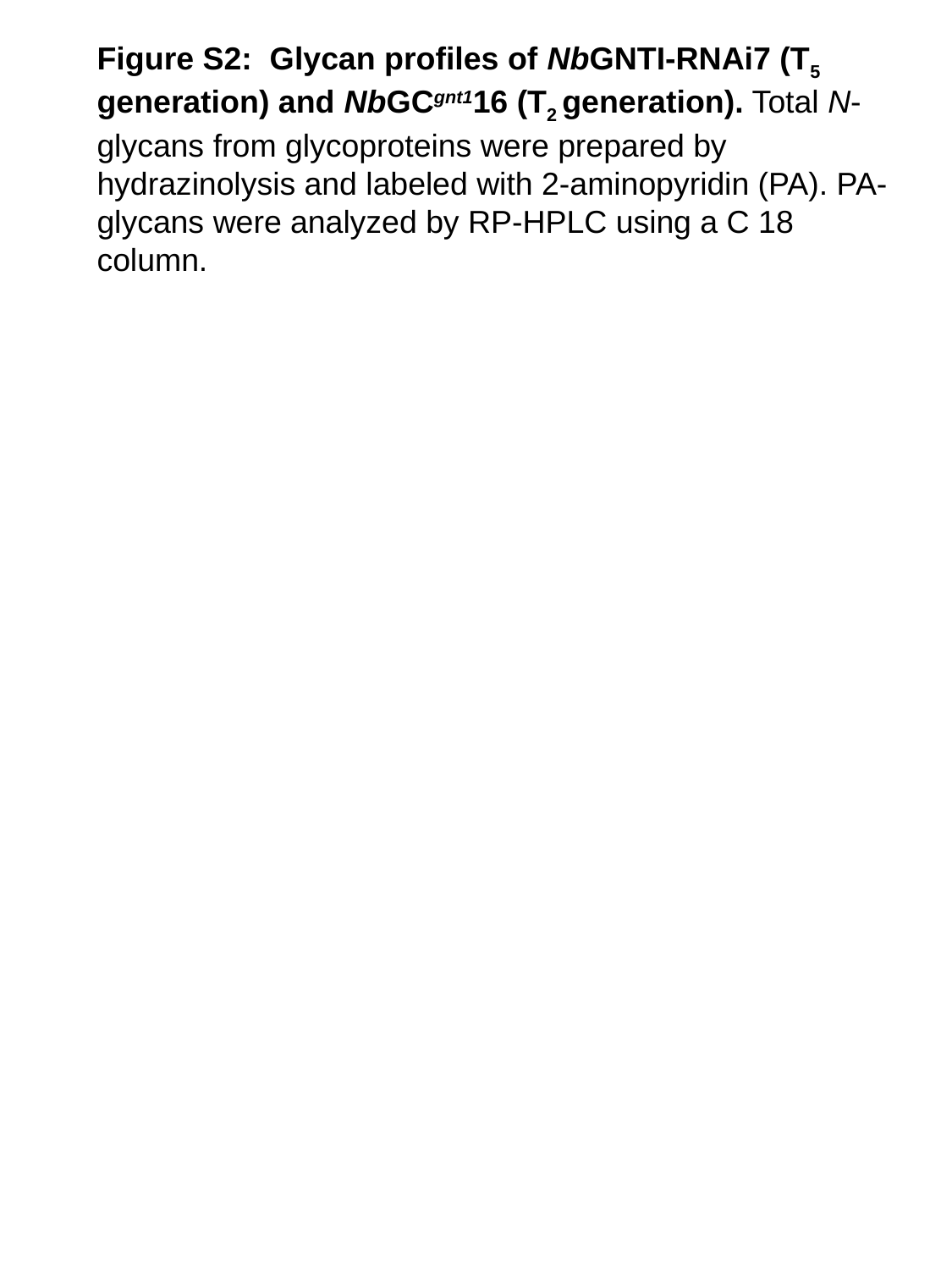

Figure S2: Glycan profiles of NbGNTI-RNAi7 (T5 generation) and NbGCgnt116 (T2 generation). Total N-glycans from glycoproteins were prepared by hydrazinolysis and labeled with 2-aminopyridin (PA). PA-glycans were analyzed by RP-HPLC using a C 18 column.

## Slide 7
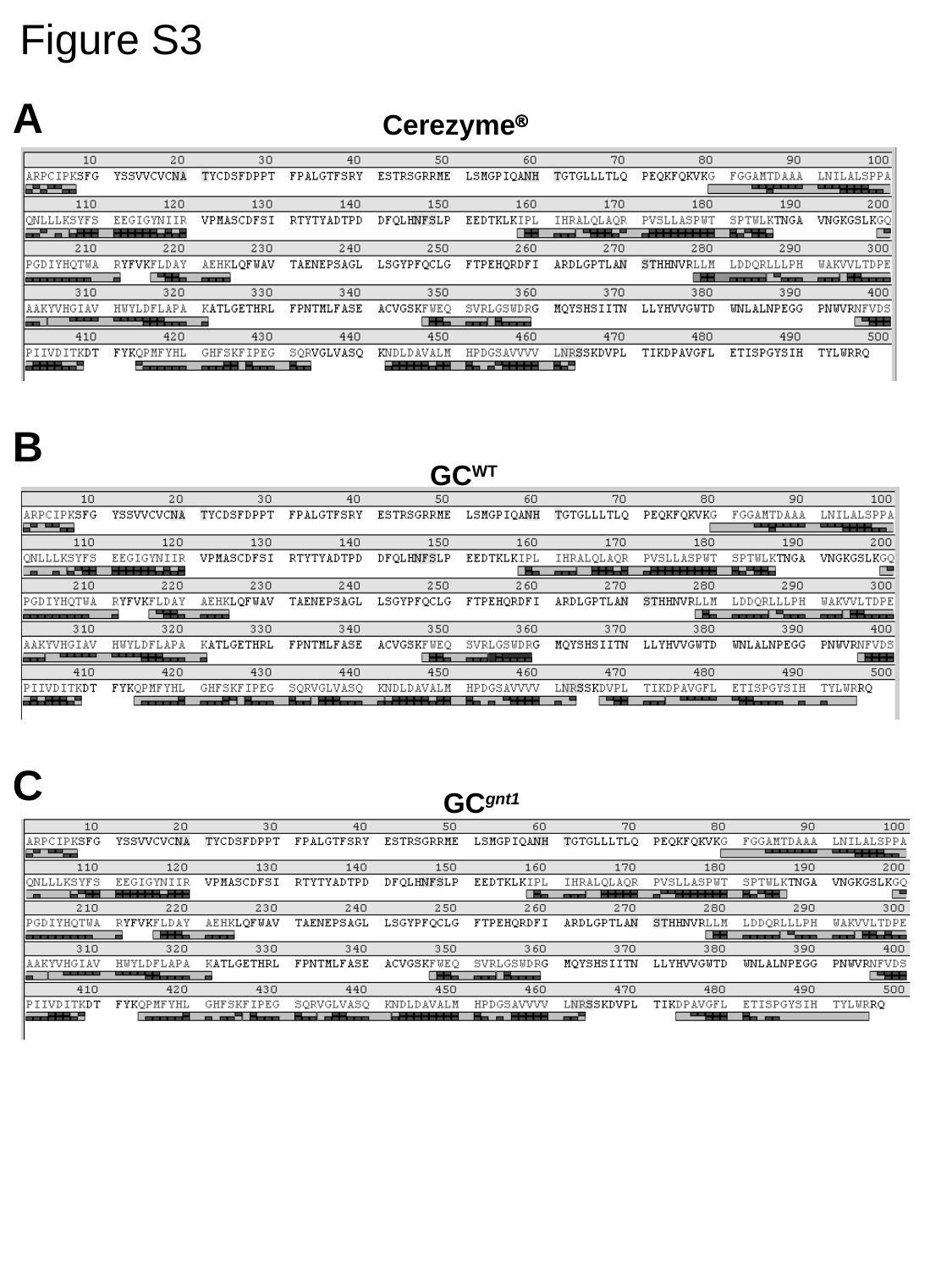

Figure S3
A
Cerezyme
B
 GCWT
C
 GCgnt1

## Slide 8
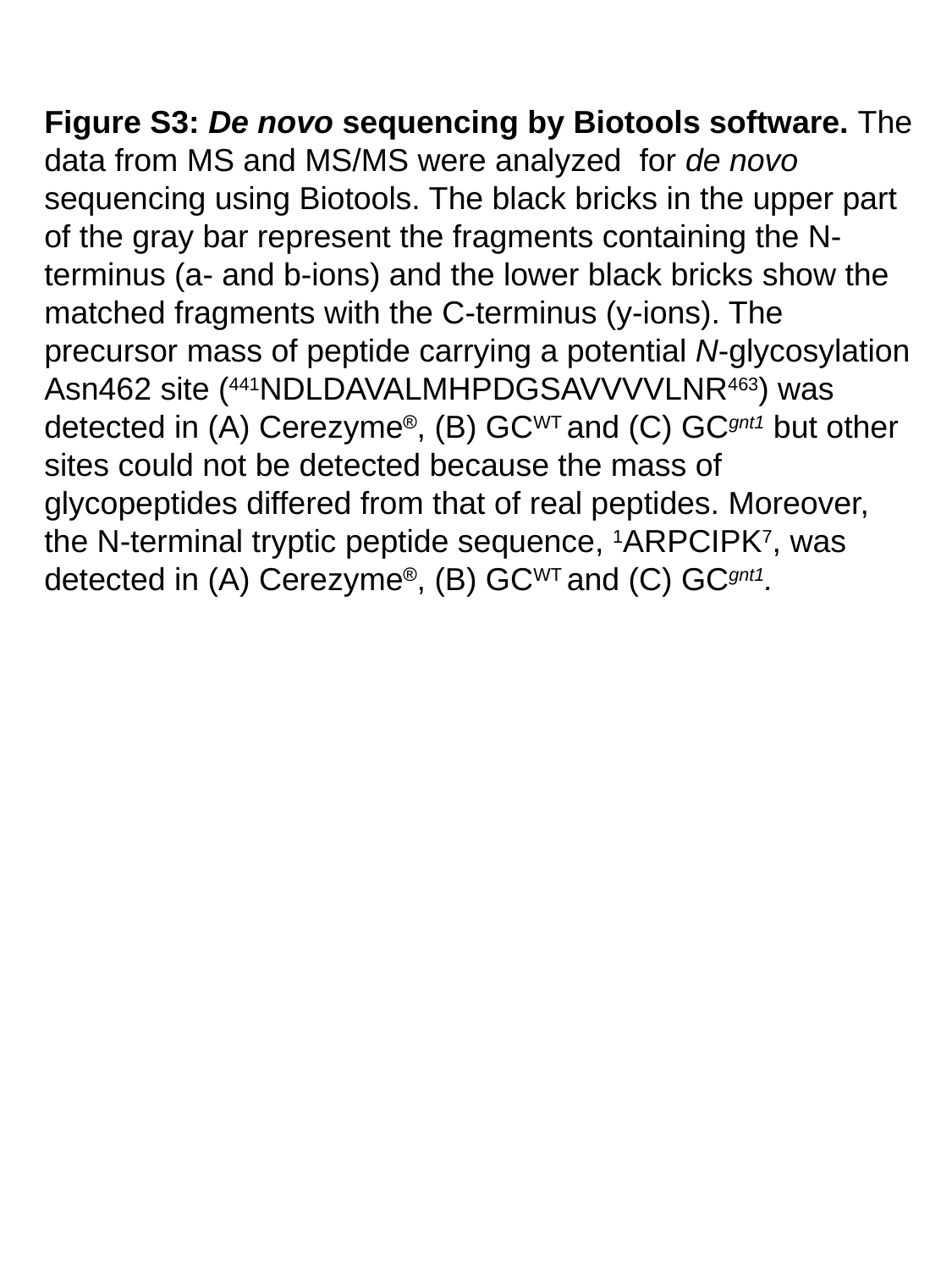

Figure S3: De novo sequencing by Biotools software. The data from MS and MS/MS were analyzed for de novo sequencing using Biotools. The black bricks in the upper part of the gray bar represent the fragments containing the N-terminus (a- and b-ions) and the lower black bricks show the matched fragments with the C-terminus (y-ions). The precursor mass of peptide carrying a potential N-glycosylation Asn462 site (441NDLDAVALMHPDGSAVVVVLNR463) was detected in (A) Cerezyme, (B) GCWT and (C) GCgnt1 but other sites could not be detected because the mass of glycopeptides differed from that of real peptides. Moreover, the N-terminal tryptic peptide sequence, 1ARPCIPK7, was detected in (A) Cerezyme, (B) GCWT and (C) GCgnt1.

## Slide 9
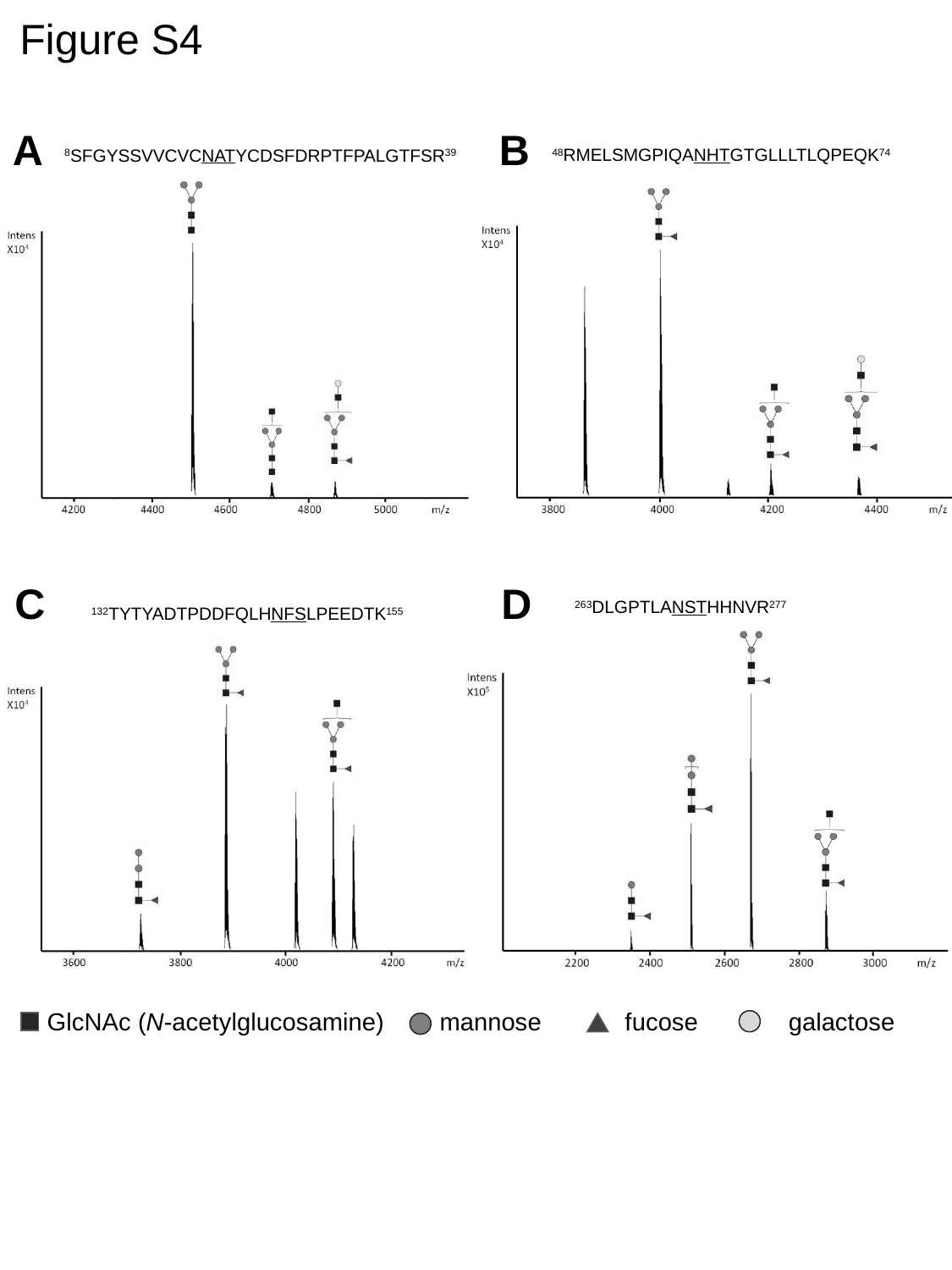

Figure S4
A
B
48RMELSMGPIQANHTGTGLLLTLQPEQK74
 8SFGYSSVVCVCNATYCDSFDRPTFPALGTFSR39
C
D
 132TYTYADTPDDFQLHNFSLPEEDTK155
263DLGPTLANSTHHNVR277
GlcNAc (N-acetylglucosamine) mannose fucose galactose

## Slide 10
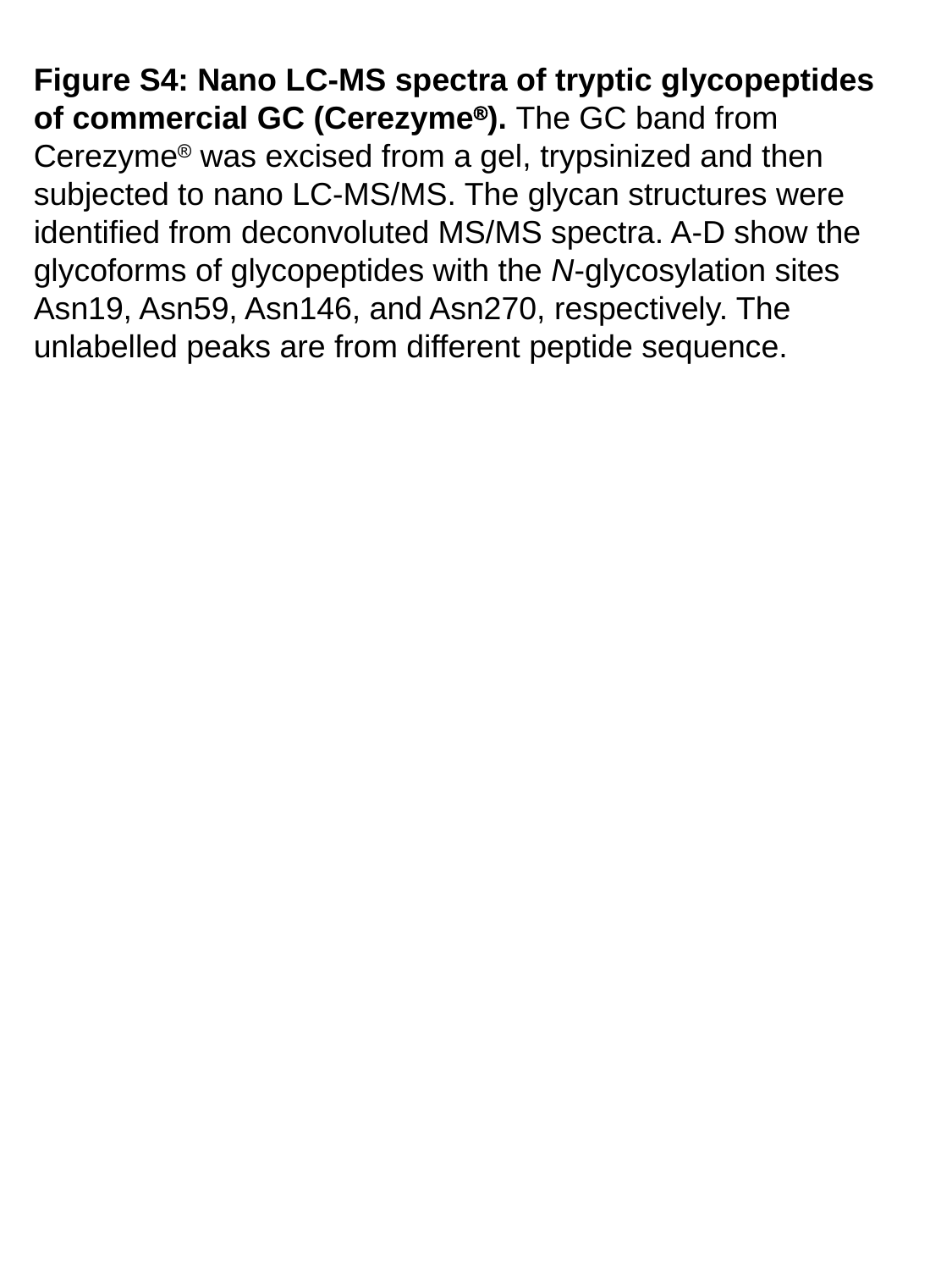

Figure S4: Nano LC-MS spectra of tryptic glycopeptides of commercial GC (Cerezyme). The GC band from Cerezyme was excised from a gel, trypsinized and then subjected to nano LC-MS/MS. The glycan structures were identified from deconvoluted MS/MS spectra. A-D show the glycoforms of glycopeptides with the N-glycosylation sites Asn19, Asn59, Asn146, and Asn270, respectively. The unlabelled peaks are from different peptide sequence.

## Slide 11
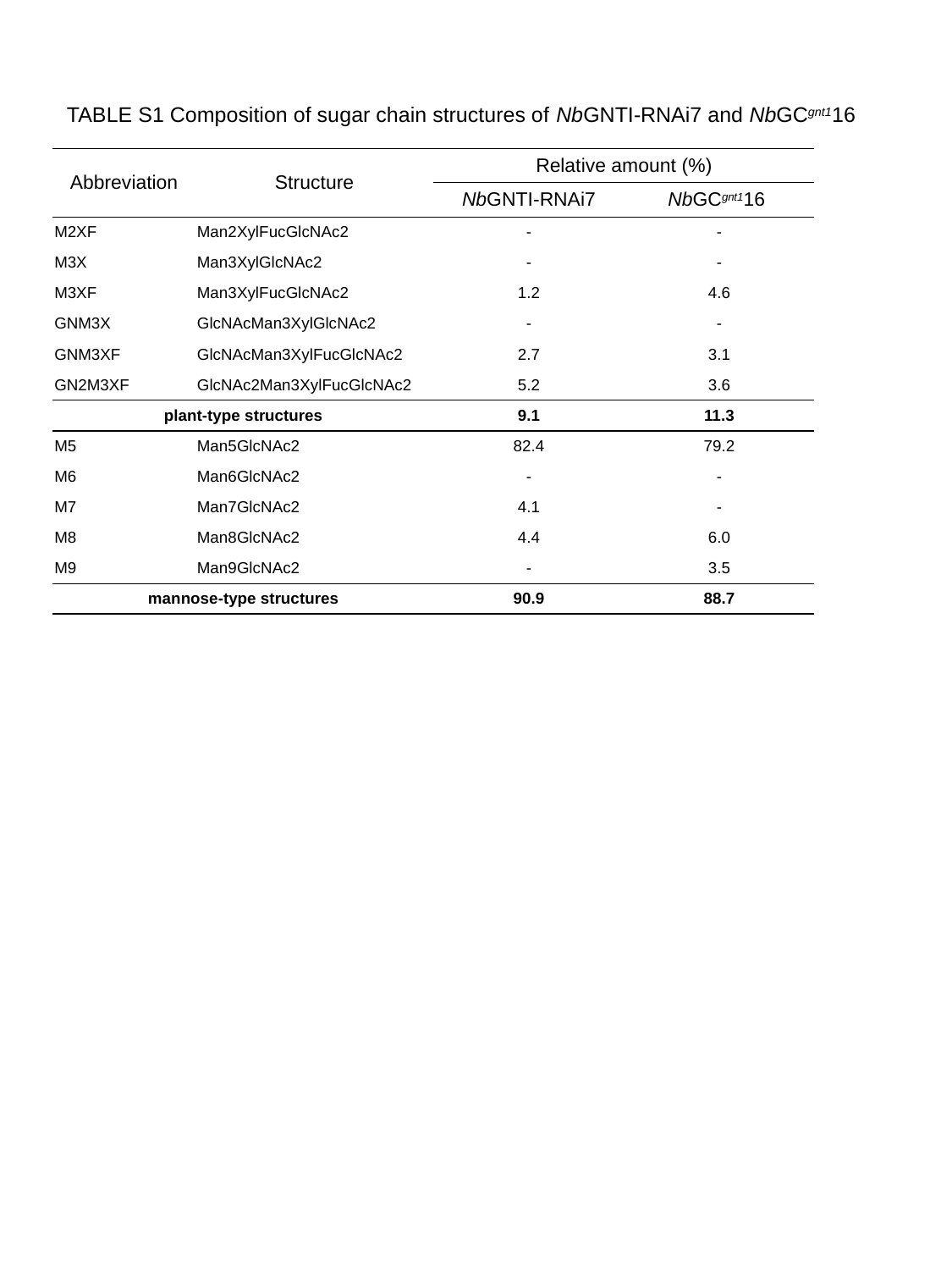

TABLE S1 Composition of sugar chain structures of NbGNTI-RNAi7 and NbGCgnt116
| Abbreviation | Structure | Relative amount (%) | |
| --- | --- | --- | --- |
| | | NbGNTI-RNAi7 | NbGCgnt116 |
| M2XF | Man2XylFucGlcNAc2 | - | - |
| M3X | Man3XylGlcNAc2 | - | - |
| M3XF | Man3XylFucGlcNAc2 | 1.2 | 4.6 |
| GNM3X | GlcNAcMan3XylGlcNAc2 | - | - |
| GNM3XF | GlcNAcMan3XylFucGlcNAc2 | 2.7 | 3.1 |
| GN2M3XF | GlcNAc2Man3XylFucGlcNAc2 | 5.2 | 3.6 |
| plant-type structures | | 9.1 | 11.3 |
| M5 | Man5GlcNAc2 | 82.4 | 79.2 |
| M6 | Man6GlcNAc2 | - | - |
| M7 | Man7GlcNAc2 | 4.1 | - |
| M8 | Man8GlcNAc2 | 4.4 | 6.0 |
| M9 | Man9GlcNAc2 | - | 3.5 |
| mannose-type structures | | 90.9 | 88.7 |

## Slide 12
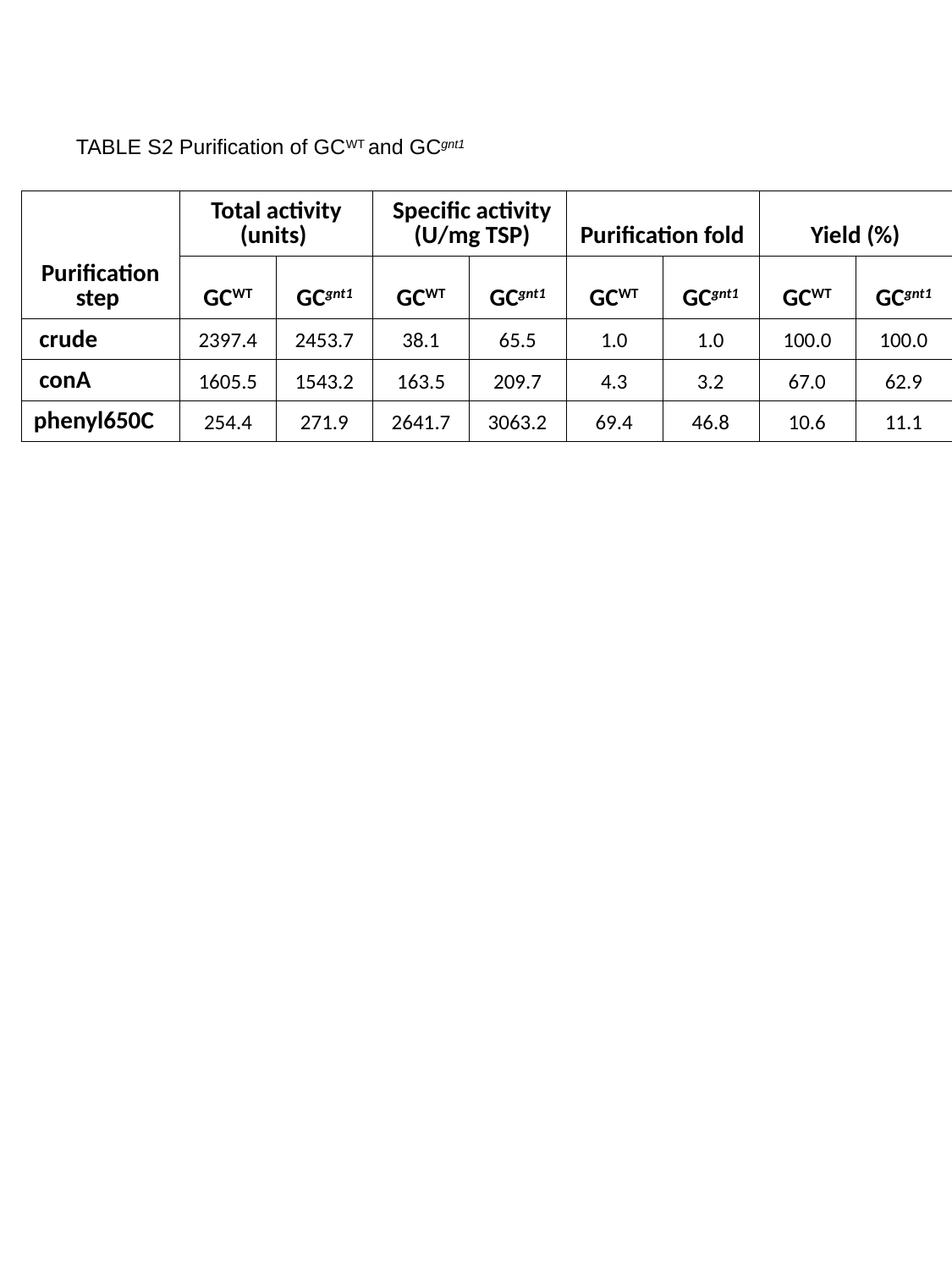

TABLE S2 Purification of GCWT and GCgnt1
| Purification step | Total activity (units) | | Specific activity (U/mg TSP) | | Purification fold | | Yield (%) | |
| --- | --- | --- | --- | --- | --- | --- | --- | --- |
| | GCWT | GCgnt1 | GCWT | GCgnt1 | GCWT | GCgnt1 | GCWT | GCgnt1 |
| crude | 2397.4 | 2453.7 | 38.1 | 65.5 | 1.0 | 1.0 | 100.0 | 100.0 |
| conA | 1605.5 | 1543.2 | 163.5 | 209.7 | 4.3 | 3.2 | 67.0 | 62.9 |
| phenyl650C | 254.4 | 271.9 | 2641.7 | 3063.2 | 69.4 | 46.8 | 10.6 | 11.1 |
